# Expert Opinion on the Efficacy and Safety of Antispasmodics with a Focus on Irritable Bowel Syndrome

**DOI:** 10.5152/tjg.2025.101125

**Published:** 2025-11-25

**Authors:** Serhat Bor, Hasan Özen, İsmail Hakkı Kalkan, Filiz Akyüz, Altay Çelebi, Dinç Dinçer, Yusuf Serdar Sakin

**Affiliations:** 1 Department of Gastroenterology, Ege University Faculty of Medicine, İzmir, Türkiye; 2 Department of Pediatric Gastroenterology, Hepatology and Nutrition, Hacettepe University Faculty of Medicine, Ankara, Türkiye; 3 Department of Gastroenterology, TOBB University of Economics and Technology School of Medicine, Ankara, Türkiye; 4 Department of Gastroenterology, İstanbul University İstanbul Faculty of Medicine, İstanbul, Türkiye; 5 Department of Gastroenterology, Kocaeli University School of Medicine, Kocaeli, Türkiye; 6 Department of Gastroenterology, Akdeniz University School of Medicine, Antalya, Türkiye; 7 Department of Gastroenterology, Acıbadem Kent Hospital, İzmir, Türkiye

## Abstract

Antispasmodics are commonly used to treat irritable bowel syndrome (IBS) symptoms. They are generally regarded as safe.However, some have been found to be ineffective or not cost-effective. Marked discrepancies in the availability and formulation of antispasmodic agents across countries—ranging from the limitation to only fundamental compounds in regions such as the United States to the marketing of diverse combination products elsewhere—have generated considerable ambiguity and debate within the field. Meta-analyses show varying results on the efficacy of antispasmodics, indicating the need for further analysis. This article aims to evaluate the effectiveness of antispasmodic drugs in relieving symptoms in IBS patients. We conclude that most drugs are considered safe and effective, with pinaverium, otilonium, and peppermint oil having meta-analyses supporting their efficacy. However, there is a lack of high-quality data for drugs like alverine, trimebutine, and cimetropium, and some drugs, such as simeticone or combinations of spasmolytic agents and simeticone, have insufficient research data. Clinicians should prioritize evidence-based medicine when selecting antispasmodic agents.

## Introduction

Irritable bowel syndrome (IBS) is a disease that is frequently observed all over the world and is characterized by predominantly recurrent abdominal pain (RAP) in addition to bloating and defecation disorders.^1^ Its pathophysiology is not fully understood, but altered gut motility and Gut–Brain Axis Regulation, Low-Grade Inflammation and Immune Activation, Altered Gut Microbiota, Food Sensitivities & FODMAP Fermentation, increased pain sensitivity have been contributed for IBS symptoms.^2^^,^^3^

Antispasmodics constitute the drug group with different mechanisms of action, some known like direct relaxation of smooth muscles (mebeverine, peppermint oil (PO)), blocking cholinergic receptors (hyoscine butyl bromide (HBB), hyoscyamine, pirenzepine), blocking calcium (Ca2+) channels (alverine citrate (AC), pinaverium bromide (PB), otilonium bromide (OB) and some unknown or mixed action mechanisms. They are preferred in many diseases with abdominal pain and spasm, especially IBS and in some cases functional diarrhea and functional abdominal bloating and distension. Evaluation of the present antispasmodics revealed that some of them failed to exhibit superiority over placebo and only imposed an economic burden on patients. For that reason, it is important to analyse the current data in those widely used medications in terms of mode of action, efficiacy and safety.

In the current literature, different results were obtained in meta-analyzes related to the efficacy of antispasmodics, and we found that the studies evaluated in the meta-analyzes were different, and the results were variable^4^^-^^13^ (Tables 1 and 2) because each meta-analysis covers different studies with different inclusion and exclusion criteria.

Therefore, this article aims to present a comprehensive evaluation of the efficacy of pharmacological treatments on symptom management in patients with IBS, through the analysis of clinical studies investigating the effectiveness and safety of antispasmodic agents.

## Materials and Methods

In order to determine the articles related to the effectiveness and safety of antispasmodics, a literature search was conducted with the keywords shown in the Table 3. The research was performed in PubMed and Google Scholar with determined keywords. Available studies were shared and analyzed in groups by the authors. Only full text and English articles were used. As a result of evaluating the studies on the efficacy of the drugs, the quality of evidence for each drug was determined, and the drugs were subsequently classified into four categories. While Grade A shows high quality evidence, Grade D has been evaluated as very low quality of evidence (Table 4). All the included trials in this study were shown in Table 5.

## Drugs

### Hyoscine-N-Butylbromide

**Figure 1.**
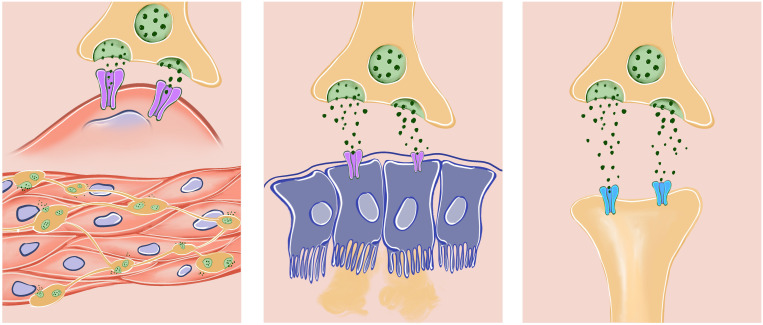
Pharmacological targets of HBB. Muscarinic receptors participate in smooth muscle contractions (left) and epithelial secretion (middle) whereas nicotinic receptors are involved in neural communication between neuron of the enteric nervous system (right).^16^

**Mode of action: **Hyoscine-N-Butylbromide is a quaternary ammonium derivative which is extracted from the leaves of the Duboisia tree found mainly in Australia. It is only partially absorbed (8%) following oral administration and the systemic availability was found to be less than 1%. It does not pass the blood-brain barrier and plasma protein binding is low. Most of the drug is metabolized in the liver, with the half-life of the terminal elimination phase being approximately 5 hours. Approximately half the metabolites are excreted in the urine. Hyoscine-N-Butylbromide blocks the action of acetylcholine at parasympathetic sites (both muscarinic and nicotinic receptors) in smooth muscle, and in secretory glands. It causes decreased motility of the gastrointestinal tract (Figure 1). This spasmolytic and smooth muscle relaxant effect helps for the treatment of abdominal pain.^14^^,^^16^

**Clinical efficacy: **There are limited studies about the therapeutic effect of HBB for IBS patients in the literature. Most of the existing studies have investigated its effect on abdominal pain. In the limited number of studies evaluating its effect on IBS, diagnostic criteria were based on Rome II or were not specified at all.

Hyoscine-N-Butylbromide effect was compared with lorazepam and ispaghula husk in a double-blind controlled study in patients with IBS and showed similar effect to placebo.^17^ Khalif et al evaluated 118 patients with IBS, diagnosed by Rome II criteria and 45 healthy individuals. They compared oral HBB 3x20 mg/day (n=37), a HBB suppository 30 mg/day (n=21), oral drotaverine 3x80 mg/day (n=30), calcium gluconate tablets 3x1/ day (n=16) as a control for oral agents, or calendula suppository/day (n=14) as a control for those who received a suppository. Hyoscine-N-Butylbromide significantly reduced abdominal pain scores among diarrhea dominant IBS (IBS-D) patients. No significant differences between placebo and other groups were evident. Meanwhile, HBB led to a significant augmentation of the rectal threshold for discomfort/pain in 58% of IBS-D subjects. Hyoscine-N-Butylbromide also decreased rectal sensitivity in IBS-D patients (*P* < .05).^18^ Lacy et al evaluated the on-demand effect of HBB in 175 patients who had abdominal pain associated with cramping. This study showed that abdominal pain significantly reduced after the HBB treatment [-0.7 (95%CI -1.3 to -0.1), *P* = .016 for episode 1 and -0.6 (95%CI -1.2 to 0.0, *P* = .051) for episode 2]. Authors concluded that HBB is effective for the treatment of RAP associated with cramping.^19^ Another study (four-arm, double-blind) included total 1637 patients and evaluated the efficacy and tolerability of oral HBB, paracetamol and their fixed combination against placebo in patients with crampy RAP . According to the Verbal Rating Scale, symptom reduction was statistically significant in all active treatment groups—HBB, paracetamol, and the combination—each showing a decrease of 0.7, compared with a decrease of 0.5 in the placebo group (all *P* < .0001). They concluded that HBB, paracetamol and their fixed combination are effective in crampy RAP and well tolerated if used three times daily continuously for 3 weeks.^20^

**Safety:** As might be expected based on the action of the drug, potential adverse event(s) (AE(s)) are numerous, and involve almost all organ systems. The most common are ocular (blurred vision, photophobia, increased intraocular pressure), respiratory (dry nose, bronchospasm), cardiovascular (tachycardia, palpitation), gastrointestinal (constipation, xerostomia), renal (urinary retention).^15^ On the other hand, HBB appeared to be relatively safe and well tolerated. The incidence of typical systemic anticholinergic, atropine-like AEs are very low and similar to that of placebo. A specific pattern of anticholinergic AEs like dry mouth could not be detected. This is because HBB is poorly absorbed and has a bioavailability of less than 1%.^21^ Mueller-Lissner et al showed that HBB (n=415) was well tolerated and had similar AEs as placebo (n=414) (16% vs 11%).^20^ Another study also confirmed these results and they revealed that AEs were similar between the HBB and placebo treatment groups (10.2% and 10.3%, respectively). The most frequently reported AEs were abdominal pain, diarrhea and joint sprain in the HBB group. They did not report any serious AE.^19^ Given that the reported safety outcomes are derived from short-term studies, the long-term use of this drug may not be advisable. There is insufficient information about HBB in pregnancy and lactation. Therefore, use of HBB is not recommended during pregnancy and breastfeeding. 

As a summary, most of the limited studies investigated the effect of HBB in short-term period and they focused only abdominal pain or intestinal colic symptoms. On the other hand, potential AEs should be considered in all patients. Considerable heterogeneity was detected between individual trials. Therefore, it is difficult to say that HBB can be included in IBS treatment options.

**Conclusion:** Hyoscine-N-Butylbromide is not effective in IBS treatment (Grade C). Long term safety is not clear. There is no data in the literature concerning the safety of HBB when used in pregnancy or lactation.

### Cimetropium Bromide

**Mode of action:** Cimetropium bromide (CB) is a quaternary ammonium antimuscarinic with peripheral effects similar to those of atropine. It is a muscarinic receptor antagonist (anticholinergic, parasympatholytic agent) and functions as a gastrointestinal antispasmodic thought to act selectively on the gut by antagonizing acetylcholine at muscarinic receptors on smooth muscle. Unlike other antimuscarinic agents, it has minimal effects on muscarinic vascular receptors and therefore has none of the serious AEs of other antimuscarinic compounds.^22^^-^^24^

**Clinical efficacy:** Some old and limited studies evaluated the effect of CB in IBS. Piai et al. demonstrated a significant improvement in abdominal pain in patients with IBS in a double-blind, placebo-controlled trial with a three-month duration. This improvement observed in second month.^25^ Ferrari et al evaluated CB effect in IBS patients for short term period. Forty patients with IBS were included in this study. They randomly allocated to treatment with OB (20 mg, 3×1/day) or CB (50 mg, 2×1/day) in a double-blind trial lasting for 6 weeks. They revealed that statistically significant decreases in severity of abdominal pain and subjective scores for bowel habits were obtained in both groups.^26^ A randomized controlled (RCT) study in patients with IBS also reported the beneficial effect of CB. Abdominal pain scores had decreased an average of 16% in the placebo group and 87% in the CB group (*P *< .01) at the end of the therapy. Authors concluded that long-term treatment of IBS with CB significantly improves abdominal pain and global symptoms.^27^ After these posivite results for IBS, Passaretti et al investigated the effects of CB on gastrointestinal transit. Forty patients, divided according to their initial total gastrointestinal transit times and presenting symptoms, were treated with CB 50 mg three times daily or placebo for 1 month according to a double-blind, parallel group design. They evaluated total intestinal transit time by radio-opaque markers (plain X-ray films of the abdomen taken every 24 h for 4 days). Cimetropium bromide significantly (*P *< .01) shortened the whole gut transit time in patients with prolonged transit time (80.8 ± 4.0 h before vs 60.8 ± 6.7 h after treatment) and improved the global clinical condition significantly compared with placebo (*P* = .029). This study showed that CB can be useful in the treatment of constipation dominant IBS.^28^

In another study with IBS patients seventy consecutive outpatients were given CB (50 mg, three times daily) or placebo according to a double-blind, randomised, parallel groups design. Abdominal pain score decreased by 40, 66, 85% in the CB group, at the end of the first, second and third months respectively, compared with 26, 32 and 52% reductions among controls (*P* = .0005). At the end of treatment there was a 86% reduction in the number of abdominal pain episodes per day in the CB group compared with 50% in the placebo group (*P* = .001). At the end of the study 89% of the patients treated with CB considered themselves as globally improved as opposed to 69% in the placebo group (*P* = .039). The results of this study showed that CB is effective in relieving pain in patients with IBS. On the other hand, this study did not confirmed the CB effect on motility.^29^

A meta-analysis (23 RCTs) of smooth muscle relaxants in the treatment of IBS showed that myelorelaxants are superior to placebo. 

In this meta-analysis, four trials about CB were included.^5^ In cochrane analyses, no statistically significant effect was found for improvement of abdominal pain (SMD 1.08; 95%CI 0.73 to 1.43; 146 patients).^27^^-^^29^ On the other hand, a statistically significant effect for improvement of global assessment was found for CB/dicyclomine (relative risk (RR) 1.88; 95%CI 1.04 to 3.42; 255 patients).^9^

**Safety:** Piai et al reported only few AEs in their study. Two patients on CB complained of dry mouth and 3 patients reported dizziness.^25^ However Ferrari et al reported some AEs with CB. The only statistically significant differences between treatments were in nondigestive symptoms (asthenia, palpitations, tremor, headache, etc).^26^ In another study, AE rate was 48% on CB group in the long-term period. The most frequently reported AEs associated with the drug were dry mouth and sleepiness.^27^ Dobrilla et al reported slight dry mouth in 6 patients taking CB.^29^ There is no data about safety during pegnancy and lactation.

In summary, a limited number of older studies reported some favorable outcomes; however, these findings are constrained by substantial heterogeneity across trials. The diagnostic criteria for IBS varied among studies, further limiting comparability and generalizability. In addition, long-term data indicated a higher incidence of anticholinergic AEs in the CB group. Consequently, more robust and contemporary evidence on both efficacy and safety is required before CB can be recommended for the management of IBS.

**Conclusion:** Cimetropium bromide seems to be effective in the treatment of IBS based on 5 heterogenous, old RCTs (Grade C). Consistent efficacy was observed in most studies in 4 weeks. According to 5 variable designed/duration studies, CB has mild AE related to anticholinergic effect. There is no data about safety during pregnancy and lactation.

### Peppermint-oil

**Figure 2.**
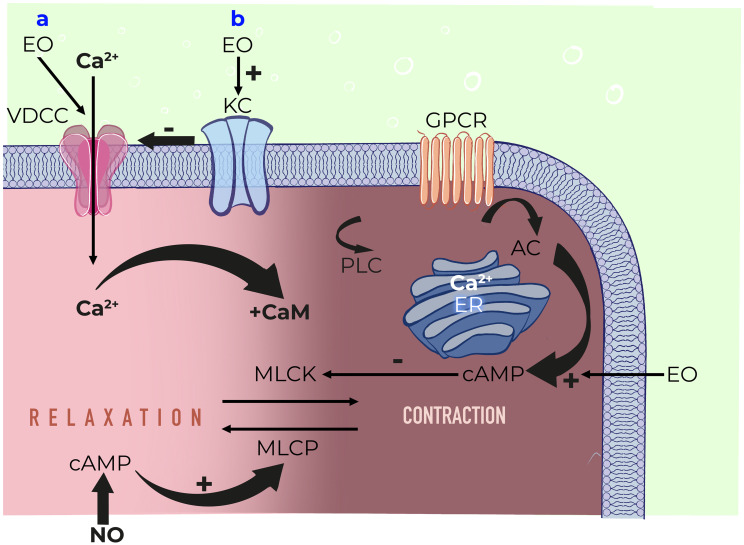
Main mechanisms of antispasmodic effect of essential oils.^42^ a. Inhibition of voltage-dependent calcium channels; b. Modulation of potassium channels; c. Modulation of intracellular cAMP (Adenylyl cyclase, AC; Calmoduline, CaM; Cyclic adenosine monophosphate, cAMP; Cyclic guanosine monophosphate, cGMP; Endoplasmic reticulum, ER; Essential oil, EO; G-protein coupled receptors, GPCR; Myosin-light chain kinase, MLCK; Myosin-light chain phosphatase, MLCP; Nitric oxide, NO; Phospholipase C, PLC; Potassium channel, KC; Voltage-dependent calcium channel, VDCC).

**Mode of action:** Peppermint is a perennial flowering plant that grows throughout Europe and North America. Peppermint oil is extracted from the mentha plant and is a complex mixture of terpenes, which can vary with growing conditions, time of harvest, and method of distillation.^30^ L-menthol is the principal component of PO, accounting for 35–50% of the compound with more than 90 other minor components making up the remainder. Peppermint oil products are, enteric-coated capsules originally developed in the 1970s. The specifications of the PO included in the formulation used in a trial were established to ensure a high level (47.5 ± 2.5%) of free L-menthol. The specifications for the active formulation included the level of PO (90 mg) and free L-menthol (41.5 mg) per capsule. A standard dose of 2 capsules contains approximately 83 mg of L-menthol, designed to release over 4 h after exiting the stomach. A pharmacokinetic (PK) study of a single immediate-release, 100-mg dose of L-menthol in healthy adults detected only menthol glucuronide in plasma or urine, while no free menthol was detected.^31^ Peppermint oil and its active ingredient, L-menthol, are known to provide smooth muscle calcium channel antagonism (Figure 2). Peppermint oil induces intestinal smooth muscle relaxation and desensitization of nociceptive nerve afferents.^32^ It normalizes orocecal transit time^33^ and has kappa opioid agonism.^34^ It has also anti-infective^35^ and anti-inflammatory effects.^36^ In addition to the antispasmodic effect, PO has been shown to inhibit 5-HT3 in the human colon.^37^ All of these proposed mechanisms of action make PO an attractive possible pharmacotherapeutic agent for IBS.

Enteric-coated PO capsules can be ruptured in the stomach and have been associated with heartburn and nausea.^38^ Additionally, delayed release of L-menthol has been associated with burning, stinging or pricking as well as cold sensation.^39^

Weerts et al. compared the PK parameters of the currently available small-intestinal–release oral formulation with those of a novel ileocolonic-release oral formulation. Another PO formulation designed to promote sustained release of PO in the small intestine. This controlled release is designed to overcome unpredictable delivery and tolerability issues with older PO technology and is implemented by converting PO into a solid-state matrix with microcrystalline cellulose as a spherical core, designed to release over 4 h in a simulated intestinal medium. The microspheres have an average diameter of less than 1,5 mm to allow rapid transit through the pylorus irrespective of the digestive stage of the stomach.^40^ Weerts et al. compared the PK parameters of the currently available small-intestinal–release oral formulation with those of a novel ileocolonic-release oral formulation. This study showed that the ileocolonic release of PO has a significantly delayed peak menthol glucuronide concentration, and therefore more distal intestinal release of PO. The ileocolonic release may enhance therapeutic efficacy as it results in increased exposure to the colonic mucosal afferent nerves and decrease AEs.^41^

**Clinical efficacy:** Two old studies reported that PO in enteric-coated capsules appears to be an effective and safe preparation for the symptomatic treatment of the IBS.^43^^,^^44^ Few years later, Nash et al published negative results with PO in IBS patients.^45^

Liu et al evaluated the efficacy and tolerability of an enteric-coated PO formulation (Colpermin), in an RCT. A total of 110 IBS patients (66 men and 44 women), aged 18 to 70 years, were enrolled in the study (49 received placebo and 52 received colpermin). Abdominal pain, abdominal distension, stool frequency, borborygmi and flatulance are significantly reduced in PO group comparing with placebo (79% vs 43%, 83% vs 29%, 83% vs 32%, 73% vs 31%, 79% vs 22% respectively).^46^ 57 patients with IBS according to the Rome II criteria, were treated with PO (enteric-coated capsules 2×2/day or placebo) for 4 weeks in another RCT. A 4 weeks treatment with PO improved abdominal symptoms in patients with IBS.^47^ In an RCT conducted among 90 outpatients with IBS, participants received Colpermin or placebo three times daily for eight weeks. The severity of abdominal pain was also reduced significantly in the Colpermin group compared to controls at the end of therapy (42% vs 22%). Furthermore, Colpermin significantly improved the quality of life.^48^ Cash et al evaluated the effect of triple-coated

microspheres of solid state, highly purified PO in IBS patients (subjects had to meet Rome III criteria for IBS-M or IBS-D). This RCT showed that at 4 weeks, PO (180 mg/day) was associated with a 40% reduction in the total IBS symptom score from baseline (mean change -1.16, SD ± 0.807), superior to the 24.3% decrease (mean change -0.70, SD ± 0.737) observed with placebo (*P* = .0246). These beneficial effects were additionally observed within the first 24 hours. They confirmed the safety of PO and concluded that it produces rapid symptom improvement in non-constipated patients with IBS. ^40^

Mosaffa-Jahromi et al reported that symptom relief rate was 75% of patients in the anise oil group, 35% in the placebo group and 52.5% in PO group (*P *< .001).^49^

A recently published RCT of patients with IBS, showed negative results for primary outcome of abdominal pain at the end of 8 weeks PO therapy. This is the first trial that enrolled 190 IBS patients diagnosed according to the recent Rome IV criteria. This study compared small-intestinal-release PO and ileocolonic-release PO with placebo. The proportion of abdominal pain responders did not differ significantly between groups: 46.8% in small-intestinal-release PO group (Odds ratio (OR), 1.68; 95%CI, 0.80 to 3.51; *P* = .170; number needed to treat (NNT), 8.1) and 41.3% in ileocolonic-release PO group (OR, 1.39; 95%CI, 0.66 to2.90; *P* = .385; NNT, 14.5), compared to 34.4% in the placebo group.

However, the small-intestinal-release PO produced greater improvements than placebo in secondary outcomes of abdominal pain (*P* = .016), discomfort (*P* = .020), and IBS severity (*P* = .020).^50^

A meta-analysis by Ford et al., including four trials with 392 IBS patients, reported that the number needed to treat (NNT) with PO to prevent persistent symptoms in one patient was 2.5 (95%CI: 2.0 to 3.0). 52 of 197 (26%) patients randomised to PO had persistent symptoms compared with 127 of 195 (65%) receiving placebo (RR: 0.43, 95%CI: 0.32 to 0.59; Figure 4). Statistically significant heterogeneity detected between studies (I2=31.1%, *P* = .23).^7^

In Cochrane meta-analysis, PO was shown to have a statistically significant effect on the improvement in global assesment (RR 2.25; 95%CI 1.70 to 2.98; 225 patients). A statistically significant effect for improvement of IBS symptom score was found for PO (RR 1.94; 95%CI 1.09 to 3.46; 269 patients).^9^

On the other hand, PO and placebo both showed clinically meaningful improvement in IBS symptoms in one RCT.^51^

Another meta-analysis reported that PO was superior to placebo for the treatment of IBS, but AEs were more frequent, and quality of evidence was very low.^52^

**Safety:** Pulegone and menthofuran normally appear in PO in doses of between 1–9% and less than 4.0% and could potentially harm chronic PO users. European Medicines Agency states that no confirmed cases of liver damage due to PO usage have been reported. Committee on Herbal Medicinal Products reported public statement on the use of herbal medicinal products containing pulegone and menthofuran in 2005. They do advise against the continuous use of PO for longer than 3 months.

Old studies with PO reported higher AE and discontinuation rates during the therapy because of vomiting, nausea and heartburn.^43^^-^^46^ Ford et al reported that 5 AEs occurred among 174 patients assigned to PO compared to no AEs in 171 patients receiving placebo.^7^ Cash et al reported, only 3 AEs (PO group: 1; placebo group: 2) which consisted of flatulence, dyspepsia, and gastroesophageal reflux symptoms.^40^

In another study, AEs were reported 26.31% in the Colpermin group and 2.56% in the placebo group. These patients complained more often (>10%) of heartburn or dyspepsia, muscle tremor, ataxia, anorexia and nausea. After 2 weeks of follow-up, 5 patients (15.15%) had withdrawn in the Colpermin group. Anorexia, burning sensation in mouth and halitosis were among the most common complaints of patients in the Colpermin group.^49^

Weerts et al reported that the mean AE rate was higher in the PO group (placebo 2.78 vs small-intestinal-release PO 4.26 vs ileocolonic-release PO 4.45).

Although upper gastrointestinal AEs (such as gastroesophageal reflux symptoms, belching, nausea) were indeed diminished compared with the small-intestinal-release PO, the novel formulation resulted in more severe abdominal cramping in the beginning of the treatment period. Adverse events leading to discontinuation rate was also higher in PO groups (One patient in placebo group vs 3 patients in small-intestinal-release PO group and 5 patients in ileocolonic-release PO group) (*P *< .005). Most common AEs were headache, diarrhea and abdominal cramp.^50^

There is no data about the safety of oral PO during pregnancy and lactation.

To summarize, although most studies demonstrated positive effects of PO on the primary outcome of abdominal pain relief, substantial heterogeneity was present. Two studies reported negative findings. One of these was a recent trial that enrolled IBS patients diagnosed according to the Rome IV criteria. In that study, small-intestinal–release PO showed benefit only for the secondary outcome of abdominal pain, defined as at least a 50% reduction in the worst daily abdominal pain for at least four of the eight study weeks. In addition, most published studies reported a statistically significant higher rate of AEs associated with PO therapy.

**Conclusion:** Peppermint oil seems to be effective for the treatment of IBS according to 9 heterogenous RCTs (Grade B). The majority of studies demonstrated consistent effectiveness within a 4-week period. According to nine studies with varying designs and treatment durations, PO appears to be a safe agent for the management of IBS.However, therapy discontinuation rate is higher in PO groups. There is no data in the literature concerning the safety of oral PO when used in pregnancy or lactation.

### Pinaverium Bromide

**Figure 3.**
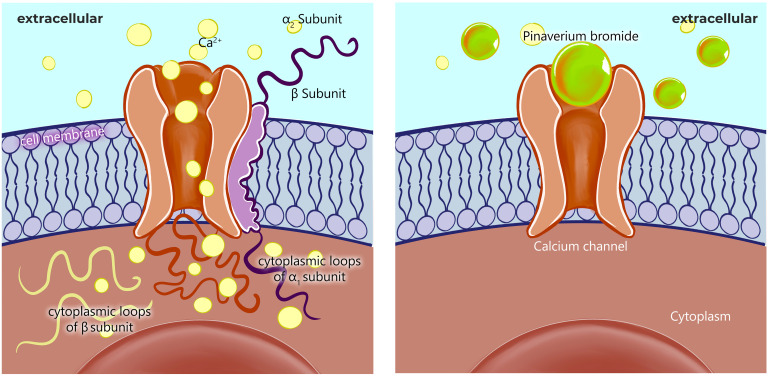
Mechanism of Action of Pinaverium Bromide.^53^

**Mode of action:** To date, among 5 different voltage dependent calcium channels, only 2 types (L and T) were reported in intestinal smooth muscle. T type was not clearly identified and it is dependent on species and tissue preperations. Pinaverium bromide as a quaternary ammonium derivative 4-[(2-bromo-4,5-dimethoxyphenyl)methyl]-4-[2-[2-(6,6-dimethyl-2-bicyclo[3.1.1]heptanyl)ethoxy]ethyl]morpholin-4-ium;bromide is a highly gastrointestinal tract selective calcium channel antagonist and it is used to treat abdominal discomfort and pain in functional lower gastrointestinal disorders due to its dual mechanism of action. It blocks *α*1-subunit of the L-type voltage-dependent calcium channels in the intestinal smooth muscle and also inhibits the contractile effect of digestive hormones and inflammatory mediators such as cholecystokinin, gastrin and substance P (Figure 3).^53^

*Clinical efficacy:* In an early pilot study including 12 patients, abdominal pain, bloating and altered bowel habits, started to ameliorate on day 4 and abnormal colonic motility patterns detected by surface electromyography were improved on day 10 of treatment with 50 mg pinaverium bromide three times daily.^54^ A continuation of this pilot study showed increased fasting and postprandial colonic motility parameters were effectively reduced and IBS symptoms (abdominal bloating, abdominal pain and stool frequency) were significantly improved by 14 day of PB therapy in IBS (n=22) patients compared to controls (n=7).^55^

A meta-analysis evaluating the efficacy of antispasmodic agents included 2 old studies assessing PB in IBS demonstrated a significant improvement in global IBS symptom assessment. In another RCT involved in this meta-analysis showed that PB was superior to placebo in the treatment of painful constipation.^56^ Similarly with the findings of abovementioned meta-analysis, PB was found to be more efficient in the treatment of IBS at the end of 28 day treatment when compared with placebo.^57^ A prospective, observational cohort study in patients with IBS, diagnosed using the Rome III criteria in four countries (Poland, Egypt, Mexico and China) assessed quality of life pre- and post-treatment (at 4 and 8 weeks) using the IBS-QoL measure. In this study, patients in China have used only PB and IBS-QoL significantly improved in patients who received PB.^58^ In two different studies PB has been compared with Chinese herbal medicine (CHM) (Tongxie formula) and found to be superior to placebo and comparable with CHM.^59,60^ In a large multicenter study, Zheng L et al showed that PB has significantly decreased pain (62.4% vs. 29.7%, *P* < .001) and improved stool consistency (53.2% vs. 20.6%, *P* < .001) at the 4^th^ week of treatment when compared with placebo in patients with IBS-D. In addition to these primary endpoints in the study, PB was also superior to placebo in terms of reducing global IBS symptoms (60.0% vs. 34.0%, *P *< .001), abdominal discomfort (53.7% vs. 32.5%, *P *< .001) and its frequency (64.7% vs. 28.2%, *P*< .001).^61^

In a recent meta-analysis including only placebo controlled studies PB has been shown to be superior to placebo for the treatment of IBS symptoms. The most striking finding in this meta-analysis was the NNT which was the lowest (NNT=4) for studies and meta-analyses of antispasmodics vs placebo in IBS.^62^ (Table 6). 

In an open label study, 91 patients with IBS-D randomized to receive 50 mg three times a day PB and 100 mg three times a day Mebeverin hydrochloride (MH). Improvement of stool consistency and global well-being were comparable between groups while the total colonic transit time was significantly prolonged only after PB treatment (21.4 +/- 15.5 vs 30.8 +/- 14.8 h, *P* < .01).^63^

A recent RCT investigated the post‑treatment therapeutic efect (PTTE) of PB in IBS-D. Pinaverium bromide was superior to placebo in terms of improvement of primary (abdominal pain and stool consistency) and secondary (pain frequency and stool frequency) outcomes. The PTTE of PB lasted 9–17 weeks which was higher than serotonin antagonists (<1 week) and similar to other antispasmodics (15 weeks). Authors of this study concluded that the stage after week 17 could be considered as IBS natural history.^64^ Clavé et al. proposed that lipophylic property of L-type calcium channel blockers may cause affinity of these drugs for colonic smooth muscle that explain the prolonged efficacy afer cessation of drug intake.^65^ In addition to this information Zheng et al emphasized that improvement of the function of the colonic smooth muscles during the treatment may explain this extended efficacy of PB beyond the treatment period.^64^

**Safety:** In 2 old randomized placebo controlled studies in IBS, PB has been shown to be comparable with placebo in term of safety.^56^ In the largest population size study, Zheng et al reported that the most common AEs associated with PB were nausea (3.7%), dizziness (3.2%), abdominal discomfort and increased blood pressure (2.3%). These rates were comparable to those observed in the placebo group.^61^

In 1997, 10 confirmed pregnant women used PB due to a dispensing error instead of Diclectin (pyridoxine/doxylamine). Fortunately all 10 women were followed-up. Nine women delivered healthy babies and the tenth one experienced a spontaneous abortion 1 week following the ingestion of PB. The duration of the ingestion of the incorrect drug varied from 1 day to 1 month before the error was detected. Half the women took the drug in the first trimester and the remaining women took the drug from 12 to 16 weeks’ of gestation. Several women experienced abdominal pain and constipation.^66^

**Conclusion:** Pinaverium bromide can be considered a first-line treatment option for IBS, as it has demonstrated efficacy in improving the full spectrum of IBS symptoms, with an acceptable profile for time to effect after treatment and a favorable safety profile in clinical practice. In addition, its use is supported by a sufficient number of published studies.

### Otilonium Bromide

**Figure 4.**
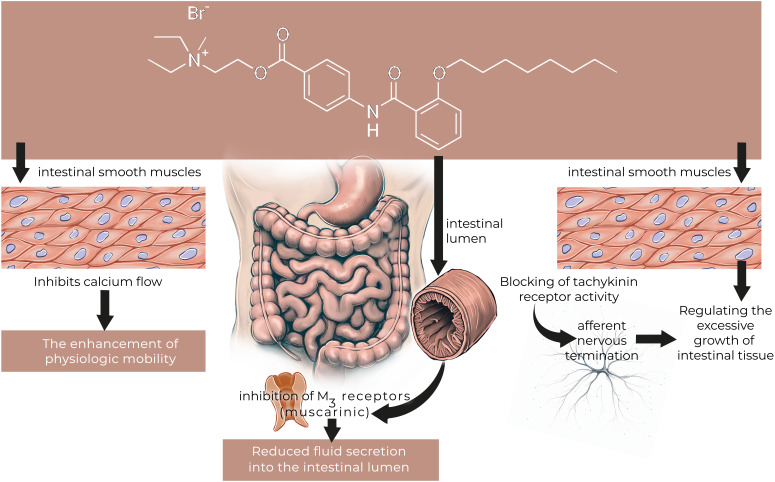
Mechanism of Action of Otilonium.^67^^-^^71^

**Mode of action:** The chemical description of OB is diethyl-methyl-[2-[4-[(2-octoxybenzoyl)amino]benzoyl]oxyethyl]azanium;bromide. In animal studies, OB a quaternary ammonium derivative that act as an antagonist of either voltage-operated Ca++ channels or selected receptors (Figure 4). Autoradiographic studies revealed that, it is poorly absorbed systemically, but effectively penetrates into the wall of the large intestine. Otilonium bromide inhibits both baseline gastrointestinal motility and stimulated motor activity by chemical or physical influences as well. It has a composite action: modifies Ca++ fluxes from intracellular and extracellular sources together with a direct action on contractile proteins; competitively inhibits muscarinic M2 receptors; and tachykinin NK2 receptors. NK2 receptors were shown to play role in the activation of sensory afferent neurons. Otilonium bromide antagonizes tachykinin receptors on the intestinal smooth muscle cells and afferent nervous terminations, thus modulating the development of intestinal hyperalgesia. Otilonium bromide, by causing inhibition of L-/T-type calcium channels, muscarinic, and tachykininergic responses concurrently, could effectively exert its pharmacologic properties.^67^^-^^71^

**Clinical efficacy:** Villagrasa M, et al compared the OB and fiber-rich diet in IBS patients. They concluded that OB induced a significant improvement of abdominal pain and distension (*P *< .001).^73^ The first large (n:72, 4 week) multicenter RCT showed that OB significantly reduced the abdominal pain, bloating and increased the pain threshold compared to placebo (*P *< .02). But frequency of bowel movements did not change.^74^

In another large study, 378 patients with IBS were randomized to receive OB 40 mg, three times daily or placebo for 15 weeks. The reduction in the number of abdominal pain episodes was significantly higher in the OB group (55.3% vs 39.9%; *P *< .01) than in those taking placebo as was the severity of abdominal distension (42.0% vs. 30.2%; *P *< .05). The visual analogue scale of well-being revealed a significant improvement in patients taking OB (*P *< .05). The investigators’ global positive assessment was in favour of OB compared with placebo (65.2% vs 49.6%; *P *< .01). Bowel discomfort improved in both groups (*P *< .01), but without any statistically significant difference from placebo.^75^

An extended analysis of this trial showed the rate of response to treatment within 2–4 months was significantly higher in the OB group (36.9% vs 22.5%; *P* = .007). The total monthly and weekly responses to the single endpoints (intensity and frequency of abdominal pain and discomfort, meteorism/abdominal distension, severity of diarrhea or constipation and mucus in the stool) were significantly more frequent in the group treated with OB than in the placebo-treated group. This trial showed that the treated patients showed response independently of the predominant symptom. Otilonium bromide can be effective in all IBS subtypes. Also, patients with diarrhea have an additional benefit from OB treatment.^76^

Czimmer and colleagues evaluated 15 patients who met Rome II criteria for IBS by Synectics Visceral Stimulator Barostat using rapid phasic distension before and after OB treatment. The sensory threshold for first sensation, stool, pain and maximum tolerable volume and pressure were measured. Otilonium bromide treatment did not influence the thresholds for first sensation and stool. The pressure threshold of pain was significantly higher 1 week after treatment (*P* < .05), but the volume threshold of this sensation remained unchanged. The pressure (*P* < .05) thresholds for maximum tolerable volume were increased by 7 days OB treatment. These data suggest that OB enhances sensory thresholds to rectosigmoid distension.^78^

A systemic review and meta-analysis of randomised controlled trials published in 2008 concluded that OB was more effective than placebo in the treatment of IBS. In this meta-analysis, four trials evaluated OB in a total of 435 patients. Symptoms persisted in 111 of 216 (51%) patients assigned to OB compared with 155 of 219 (71%) of those receiving placebo (RR: 0.55, 95%CI 0.31 to 0.97, I2=59.5%), and a NNT of 4.5 (95%CI 3.0 to 10.0).^7^

In an RCT (n=356) the effect of OB was significantly greater than placebo in the reduction of weekly frequency of episodes of abdominal pain at the end of the treatment period (-0.90 ± 0.88 vs. -0.65 ± 0.91, *P* = .03), reduction of abdominal bloating (-1.2 ± 1.2 vs. -0.9 ±1.1, *P* = .02) and global efficacy by patient assessment (-1.3±1.1 vs. -1.0 ±1.1, *P *= .047). In the follow-up period, the therapeutic effect of OB remained greater than placebo in terms of withdrawal rate due to symptom relapse (10% vs. 27%, *P *= .009), global efficacy of treatment and relapse-free probability (*P* = .038). The effect of OB started in the 10th week of therapy.^65^

A systemic review and meta-analysis of RCTs concluded that global assessment with OB was better than with placebo (OR: 2.03; 95%CI 1.49 to 2.77), indicating that OB is more effective than placebo in treating IBS. The efficacy of OB on abdominal pain relief is better than placebo (OR: 1.84; 95%CI 1.43 to 2.36) and OR for abdominal distension/bloating was 1.38 (95%CI 1.00 to 1.91).^10^

Dose-ranging study demonstrates that OB at 40 and 80 mg can improve individual and global clinical symptoms of IBS compared to placebo over a 4-week period. The difference between the OB 40 and 80 mg was not significant.^79^

**Safety:** Two trials did not report any AEs.^72^ One mild nausea was reported in another study.^74^ Comparisons with other spasmolytic agents showed low incidence of weakness and dizziness for OB.^80^ A placebo-controlled study revealed two dropouts (prostate disturbance and dizziness) in the OB group compared to one dropout in the placebo group.^75^ Data from safety and postmarketing observations showed that OB is well tolerated and that AEs do not differ with those seen with placebo. A 10-year post-marketing surveillance reported only two AEs, both of which were urticaria.^81^ In a large study, two cases of dry mouth and one case of nausea were seen in OB group.^65^

**Conclusion:** Otilonium bromide is a safe medication that is significantly superior to placebo in global efficacy assessment, abdominal pain, and abdominal distension, with a side-effect profile comparable to placebo in patients with IBS (Grade B).

### Trimebutine Maleate

**Mode of action:** Animal studies showed that the actions of trimebutine maleate (TM) [3,4,5-trimethoxybenzoic acid 2-(dimethylamino)-2-phenylbutylester] on the gastrointestinal tract are mediated via (a) an agonist effect on peripheral µ, Κ and δ opiate receptors and (b) release of gastrointestinal peptides such as motilin and modulation of the release of other peptides, including vasoactive intestinal peptide, gastrin and glucagon. Trimebutine maleate accelerates gastric emptying, induces premature phase III of the migrating motor complex in the intestine and modulates the contractile activity of the colon. Trimebutine maleate has also been shown to decrease reflexes induced by distension of the gut lumen in animals and it may therefore modulate visceral sensitivity.^82^

**Clinical efficacy:** In two 3-day RCTs, TM was significantly more effective than placebo in relieving the symptoms of mild to moderate degrees of IBS (*P *< .001). But the study period is very short and Jadad score of these trials were 2.^83^^,^^84^ Moshal et al. showed a statistically significant improvement in abdominal pain and constipation in 20 IBS patients with both TM and placebo after 4 weeks, but only with TM after 8 weeks.^85^ Fielding did not find any difference between TM and placebo in the treatment of IBS in an RCT.^86^

A meta-analysis of four studies involving 116 patients utilized two statistical methods. The Peto method revealed that global improvement was significantly superior in the TM group than in the placebo group, with an OR of 3.45 (95%CI 2.03 to 5.86). Additionally, the Der Simonian method indicated a risk difference of 30% (95%CI 13% to 47%), favoring TM. However, this meta analysis included two short term studies that were not analyzed in other meta analyses.^5^

A meta-analysis examining the impact of TM included 3 trials with a total of 140 participants. Among the 70 patients who received TM, 28 (40%) experienced persistent symptoms. The difference in the proportion of patients with ongoing symptoms between the TM and placebo groups was minimal (40% vs. 39%; RR:1.08, 95%CI 0.72 to 1.61, I2=0%). Trimebutine maleate seemed to have no benefit over placebo in treating IBS.^7^ In the other systemic review and meta-analysis TM is not superior to placebo in global assessment (OR:1.27, 95%CI 0.58 to 2.79) and abdominal pain relief (OR:1.28, 95%CI 0.53 to 3.14)].^10^

**Safety:** According to Moshal et al, no AEs were observed in the 20-patient treatment group.^85^ In another study, two participants in the TM group withdrew due to AEs. The reported AEs included nausea, gastrointestinal discomfort, and tremor.^86^

**Conclusion:** The effectiveness of TM in the treatment of IBS has not been conclusively demonstrated, based on RCTs of varying designs and generally low quality (Grade C). This conclusion is derived from four RCTs with heterogeneous designs and treatment durations.

### Prifinium Bromide

**Mode of action:** Prifinium bromide (PRB) [l, l-Diethyl-3-(diphenyl methylene)-Z methylpyrrolidium bromide] is a quaternary ammonium compound with a potent antiacetylcholine action. It is an anticholinergic /antimuscarinic agent with antispasmodic and antiemetic properties.^87^

**Clinical efficacy:** There is only one clinical research in the current literature regarding the efficacy of PRB in the treatment of IBS. In a 6-week randomized controlled crossover study, 18 patients with IBS received oral administration of PRB at a dosage of 90 mg daily, taken prior to meals. Global tretament response (abdominal pain, flatulence, constipation, and/or diarrhea) showed a statistically significant difference (*P *< .01) in favor of the drug.^88^

**Safety:** Dry mouth and blurred vision were rare and mild, and none of them required treatment discontinuation.^88^

**Conclusion:** There are limited data regarding the efficacy of PRB in the treatment of IBS (Grade D).

### Dicylomine Hydrocloride

**Figure 5.**
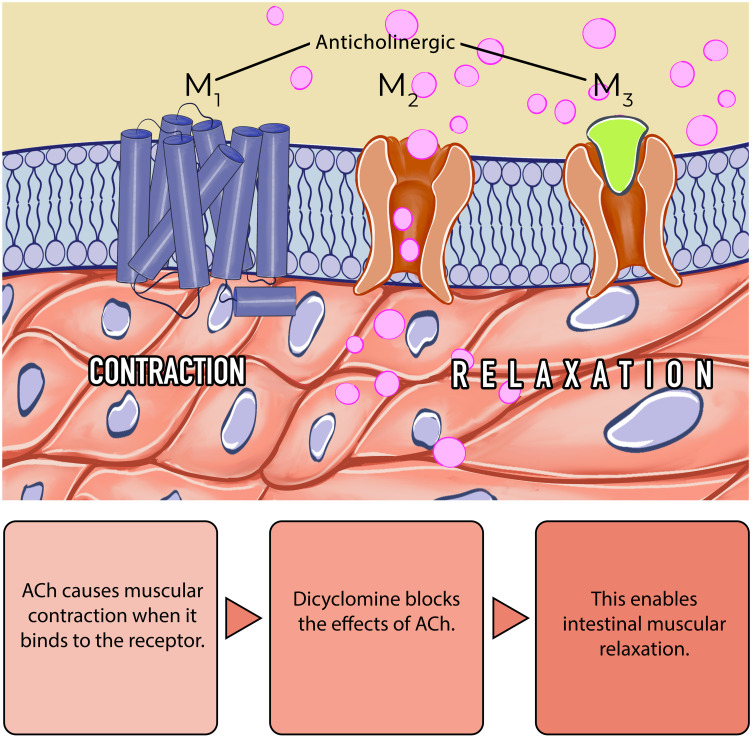
Mechanism of Action of Dicyclomine.^89^^,^^90^

**Mode of action:** Dicyclomine hydrochloride (DH) (2-(diethylamino) ethyl 1-cyclohexylcyclohexane-1-carboxylate; hydrochloride) is a carboxylic acid derivative and a selective anticholinergic with antispasmodic activity. Dicyclomine hydrochloride inhibits the binding of acetylcholine to muscarinic receptors on smooth muscle (Figure 5). Dicyclomine hydrochloride achieves its action partially through direct antimuscarinic activity of the M1 and M2 receptors, and partially through antagonism of bradykinin and histamine. Dicyclomine hydrochloride non-competitively inhibits the action of bradykinin and histamine, resulting in direct action on the smooth muscle, and decreased strength of contractions seen in spasms of the ileum.^89^^,^^90^

**Clinical efficacy:** In an RCT, 48 patients with IBS were in the DH group and 49 patients with IBS were in the placebo group. According to the physician’s global assessment of treatment, DH is superior to placebo in terms of improving all variables (abdominal pain (*P *< .001), abdominal tenderness (*P* = .003), bowel habits (*P* = .003), overall condition (**P *< .*001). Also DH is superior to placebo in patient’s self assessments of treatment (*P* = .006) and duration of abdominal pain (*P* = .047). Eleven of the patiens requiring a dose reduction were able to maintain efficacy as reflected in the physicians’s final global evaluation of the condition of the patient at the end of the study.^91^

**Safety:** Adverse events were reported in 69% (n:33) of the patients. Adverse events included dry mouth, dizziness, and blurred vision. In the 48-patient group, 12 patients required dose reduction, 7 patients withdrew from the study because of AEs.^91^

**Conclusion:** The efficacy of DH in the treatment of IBS has not been established (Grade D), and its use is not recommended due to its higher rate of AEs.

### Simeticone

Although simeticon is not an antispasmodic agent and is primarily classified as an anti-foaming compound, it has been included in this review due to its frequent use in clinical practice among patients with IBS.

**Mode of action:** Simeticone silicone latex is a defoaming agent. It acts as a detergent to reduce the surface tension of bubbles in the intestinal tract. In theory it is expected that simeticone enables abdominal gas to be expelled more easily.^92^^,^^93^

**Clinical efficacy:** There are no studies evaluating the isolated effect of simeticone on any aspect of functional bowel disorders, including IBS, abdominal bloating, and abdominal distension. Because of this lack of data it is not possible to grade the drug. Studies mainly evaluated the effect in the contest of endoscopy or videocapsule studies and visibility. A meta-analysis analyzing the efficacy of antispasmodic agents in the treatment of IBS alone or in combination,^10^ included 3 different simeticone combination studies. According to these studies, Simeticone/Alverine citrate combination were superior to placebo in improvement of IBS global assessment and pain relief^94^ while Simeticone/Pinaverium bromide combination was comparable with placebo.^95^ Another unpublished study in this meta-analysis showed that Simeticone/Pinaverium bromide combination was superior to placebo in term of improvement of abdominal distension.^96^ In a recent study, IBS patients diagnosed according to Rome III criteria were received placebo or Simeticone/Pinaverium bromide combination in a double-blind randomization for 12 weeks. Simeticone/Pinaverium bromide combination significantly decreased abdominal pain and bloating when compared with placebo at the end of the treatment period.^97^ In a multicenter, open-label study 1677 active IBS (Roma III) patients received Simeticone (300 mg)/Pinaverium bromide (100 mg) combination for 4 weeks. There were significant decreases in abdominal pain and abdominal bloating VAS scores.^98^

**Safety:** A meta-analysis evaluating the efficacy and safety of antispasmodic agents included two combination therapies containing simeticon (Simeticone/Pinaverium bromide and Simeticone/Alverine citrate), which provided safety data. These studies demonstrated that these combinations were comparable to placebo in terms of safety when used in patients with IBS.^10^^,^^94^

Although simeticone is used for infantile colic (IC), there is no data in the literature concerning safety of its use in pregnancy or lactation.

**Conclusion:** The efficacy of simeticone in the treatment of IBS has not been established (Grade D). Its role in current clinical practice remains negligible.

### Mebeverine Hydrochloride

**Figure 6.**
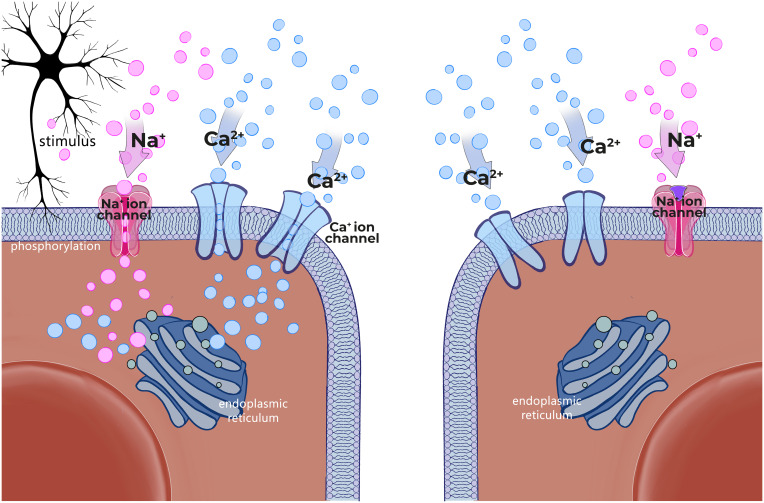
Mechanism of Action of Mebeverine: Anti-spasmodic Effect.^100^^,^^101^

**Figure 7.**
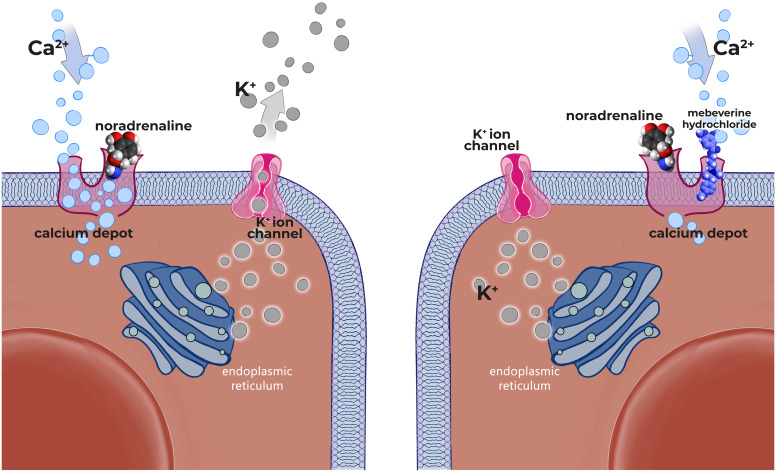
Mechanism of Action of Mebeverine: Effect on Reflex Hypotony.^100^^,^^101^

**Mode of action:** Mebeverine hydrochloride (MH) has been used as an antispasmodic for the treatment of IBS for 55 years. However, data about the mechanism of action of MH is limited. In a study investigating the effect of MH on guinea pig intestinal smooth muscle cells; it has been reported that MH inhibits the response to alpha 1 receptor stimulation and causes a decrease in sodium permeability. In this study, it was reported that the effects of MH included limitations of both depolarization and hyperpolarization in smooth muscle cells.^99^ In an RCT by Evans et al.^100^ investigating the effect of MH on small intestine motility at a dose of 135 mg, four times daily in patients with IBS and healthy volunteers; they reported that the drug had no effect on small intestinal motility in healthy controls, while it had a normalizing effect on small intestinal motility in patients with IBS. In the study published by Daly et al.^101^ it was reported that MH infusion into the sigmoid colon of healthy volunteers stopped the increase in postprandial rectosigmoid contraction. It has been suggested that MH reduces hypercontractility and, unlike anticholinergic drugs, does not affect or inhibit normal motor activity (Figures 6, 7).^100^^,^^101^ Based on the results of the above-mentioned studies, MH can be assumed to weaken hypercontractility and prevent excessive relaxation.^100^

**Clinical efficacy:** There are 6 MH RCTs published between 1965 and 2005. In 3 of these studies, MH was found to be superior to placebo.^102^^-^^104^ In the remaining 3 studies, MH was found to be similar to placebo.^105^^-^^107^ A meta-analysis of RCTs of MH in patients with IBS was published in 2010 by Darvish-Damavandi et al.^108^ The meta-analysis of studies comparing MH with a placebo revealed that, the pooled RR for clinical improvement of MH was 1.13 (95%CI 0.59 to 2.16, *P* = .705) and 1.33 (95%CI 0.92 to 1.93, *P* = .129) for relief of abdominal pain. Similar results were reported in the meta-analysis published in 2012 by Martínez-Vázquez et al^10^ in which the same studies were evaluated. In all studies evaluated in these meta-analysis, the diagnosis of IBS was made clinically, IBS subgroups were not evaluated, and the treatment follow-up period was between 4 and 16 weeks, most of which was 4 weeks. Such variations in trial designs increase the heterogeneity between studies.

In 3 studies in patients with IBS, no significant difference was found in terms of efficacy and tolerability between administering 200 mg of MH sustained-release capsule twice a day and 135 mg, three times a day.^109-^^111^

In a multicenter, randomized, 4-week clinical trial, the efficacy of the 5-HT3 receptor antagonist Ramosetron (5 µg once daily) was compared with MH (135 mg three times daily) in male patients with IBS-D diagnosed according to the Rome III criteria. At the end of treatment, the two groups demonstrated comparable response rates in terms of global improvement in IBS symptoms (37.2% vs 37.5%, respectively).^58^

In a prospective observational cohort study conducted in 4 different countries using Rome III diagnostic criteria, the effects of 4 and 8 weeks of MH or PB treatments on patients’ quality of life were evaluated using the IBS-QoL scale in patients with IBS reported that both drugs significantly improved the quality of life of the patients compared to the baseline in both the 4th week and more at the 8th week.^58^

A randomized, controlled, cross-over study involving 45 patients with IBS evaluated MH 135 mg three times daily versus AC 60 mg three times daily over a 4-week period. The findings indicated that both therapies demonstrated comparable efficacy.^112^

Evaluation of MH 135 mg three times daily versus AC 60 mg three times daily in a randomized, cross-over, 2-week trial of 45 patients with IBS demonstrated that the two treatments had similar efficacy.^84^

An 8-week RCT including 117 patients with IBS diagnosed according to the Rome II criteria compared OB 40 mg three times daily with MH 100 mg three times daily. The study concluded that both medications demonstrated comparable efficacy.^113^

In a multicenter RCT, 5 HT3 receptor antagonist Alosetron 1 mg, twice a day, was compared with MH 135 mg three times a day in 623 women with non-constipated IBS according to the Rome I diagnostic criteria. After 12 weeks of treatment, both medications showed statistically significant improvements in abdominal pain and bloating compared to baseline values. ^114^

Mebeverine hydrochloride is a well tolerated antispasmodic that has been used in the treatment of IBS for a long time. According to the meta-analysis results of the studies comparing MH and placebo treatment, the pooled RR for clinical improvement of MH was 1.13 and 1.33 for relief of abdominal pain, but the difference was not statistically significant. However, the heterogeneity between the studies is high due to the Rome criteria are not used in these studies and IBS subgroups are not taken into consideration. In drug-drug comparison studies, MH was evaluated as a reference drug in the treatment of IBS, and it was reported to have similar efficacy with other compared drugs except Alosetron. Drug-drug comparison studies with MH in the treatment of IBS are shown in Table 2.

**Safety:** In placebo-controlled studies, MH-related AEs were not different from placebo. Adverse events reported in studies are headache, nausea, dizziness, dry mouth, fatigue, constipation, diarrhea, drowsiness, palpitations,dyspnea and abdominal distension.

There is no data in the literature concerning the safety of MH when used in pregnancy or lactation.^58^^,^^108^^,^^111^^,^^115^

**Conclusion:** Mebeverine hydrochloride is not superior to placebo according to meta-analysis of RCTs (Grade C). In drug-drug comparison studies, the efficacy was similar to that of other drugs, except for Alosetron.

### Alverine Citrate

**Mode of action:** Alverine citrate is a drug that has chemical and pharmacological similarities with MH.

**Clinical efficacy:** In a randomized controlled cross-over 4-week study comparing MH 135 mg, three times a day and AC 60 mg, three times a day in 45 patients with IBS, both drugs were found to be similarly effective.^112^

In a 12-week RCT of AC 60 mg, three times a day involving 107 patients with IBS, according to modified Rome I diagnostic criteria the reduction in abdominal pain score at the end of treatment was 43.7% in the AC group and 33.3% in placebo group, although the difference was not statistically significant.^116^

In a 4-week RCT in 412 patients with IBS according to Rome III diagnostic criteria; Alverine citrate/Simeticone combination (AC 60 mg *+* simeticon 100 mg combined capsule three times a day) was found to be significantly more effective than placebo in decreasing abdominal pain/abdominal discomfort at the end of the second and fourth week.^94^

In a 6-month study in which continuous use of Alverine citrate/Simeticone combination was compared with on-demand use in patients with IBS; while IBS-QoL scores at the end of 6 months of treatment improved significantly in both groups compared to the baseline, the IBS symptom score was statistically significantly lower from the first month to the end of treatment in the on-demand treatment group compared to continuous use.^117^

Research on the effectiveness of AC for the treatment of IBS is limited. A single study reported that AC has similar efficacy as placebo in patients with IBS. Only one study reported that the combination of Alverine citrate/Simeticone was significantly more effective than the placebo.

**Safety:** In placebo-controlled studies, AC related AEs were not different from placebo.^94^^,^^116^ The AEs reported in studies are nausea, headache and upper quadrant abdominal pain.

There are four cases of AC induced acute hepatitis in the literature.

There is no data in the literature concerning the safety of AC when used in pregnancy or lactation.

**Conclusion:** There is insufficient data in the literature to recommend AC or Alverine citrate/Simeticone combination for the treatment of IBS (Grade C).

### Drotaverine Hydrochloride

**Mode of action:** Drotaverine hydrochloride (DRH) has been described in the literature as spasmolytic, which has relaxant properties acts directly on intestinal smooth muscles and has not anticholinergic side effects.^118^ In the study investigating the effect of oral DRH 80 mg, three times a day or HBB (oral or suppository) administration on rectal pain threshold and anorectal manometry parameters in patients with IBS according to Rome II; both drugs have been reported to significantly increase the rectal pain threshold in IBS-D patients without changing other anorectal manometry parameters. The authors concluded that the clinical benefits of antispasmodic drugs may be on visceral sensation rather than affecting motility.^18^

**Clinical efficacy:** A multicenter, 4-week RCT on the efficacy and safety of DRH was conducted in 180 patients who diagnosed as IBS according to Rome II criteria published by Rai et al.^118^ In this study, a DRH tablet was administered at a dose of 80 mg, three times a day, abdominal pain and stool frequency were measured weekly, and the Subject Global Assessment of Relief of IBS symptoms was evaluated. At the end of the treatment, a decrease in the score of the frequency and severity of abdominal pain was found to be significantly higher in the DRH group than placebo (78% vs 31%, respectively). This study demonstrated that after a 4-week treatment period, DRH significantly improved abdominal symptoms in patients with IBS when compared to placebo group.

Results from a 4-week randomized controlled trial evaluating DRH 80 mg three times daily versus placebo in 144 Chinese patients diagnosed with IBS according to the Rome II criteria have been reported.^119^ The primary aim of this study was to investigate the efficacy of DRH in improving abdominal pain measured using a VAS and weekly stool frequency. At the end of the treatment, improvement in pain score measured by VAS and stool frequency in patients with IBS was found to be significantly better in the treatment group compared to the placebo group. Quality of life score measured by SF 36 was found to be similar between the two groups. Unlike other studies, it was reported that improvement in abdominal pain and stool frequency was significantly better than placebo in all IBS subgroups.^18^

There are few studies in the literature on DRH, and it has been reported that it is particularly effective in improving abdominal pain and stool frequency in patients with IBS.

**Safety:** In placebo-controlled studies, DRH related AEs were not different from placebo.^118^^,^^119^ Adverse events reported in studies are headache, heartburn, flatulence, nausea, dizziness and fatigue.

There is no data in the literature concerning the safety of DRH when used in pregnancy or lactation.

**Conclusion:** While DRH appears to be beneficial for IBS treatment, the evidence is restricted to only two RCTs found in the current literature (Grade C).

### Future Perspectives

**Figure 8.**
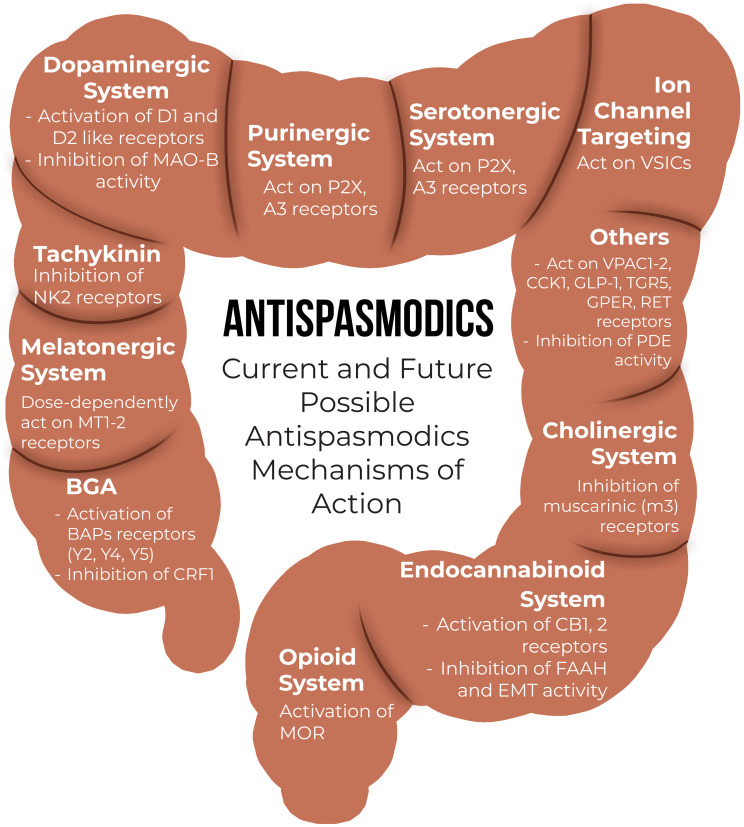
Current and Future Possible Antispasmodics Mechanisms of Action. (BAPs, Biologically active proteins; BGA, Brain gut axis; CB, Cannabinoid; CCK, Cholecystokinin; CRF1, Corticotropin-releasing factor; EMT, Endocannabinoid membrane transporter; FAAH, Fatty acid amide hydrolase; GLP, Glucagon like peptide; GPER, G Protein-coupled estrogen receptor; MAO-B, Mono amino oxidase B; MOR, Muopioid receptor; NK, Neurokinin; PDE, Phosphodiesterase; RET, Rearranged during transfection; TGR5, bile acid receptor; VPAC, VIP receptor; VSICs, Voltage sensitive ion channels.^120^

The exact pathophysiology of IBS is still not understood but evidence suggests it is most likely multifactorial with the involvement of complex neuro-immune brain-gut interactions, including disturbed gastrointestinal motility, low-grade mucosal inflammation, dysregulation of the enteric nervous system, bile acid malabsorption, altered gut flora and genetic susceptibility. Although studies are limited and available literature is not always of high quality, in the era of spasmolytic agents, many new medications are in the development phase (Figure 8). Moreover, there is a lack of clinical trials comparing different pharmacological treatments. Consequently, large clinical trials are required to evaluate and compare the efficacy of different agents, ideally using a double-blind, randomized, parallel-group design with direct head-to-head comparisons Furthermore, future studies should aim to identify predictors for treatment response, including comorbidities (e.g., anxiety and depression) and as well as potential biological markers. One important unmet need concerns the evaluation of on-demand use of these agents, which may facilitate individualized pharmacological treatment strategies for patients with IBS.

A recent article summarized the possibilities and targets of achievement of new and better spasmolytic agents.^120^ In order to have a better drug;

1. A better understanding of disorders of gut-brain interaction is needed in such a multifactorial disease,
2. Since this is a multifactorial disease, one particular drug should target multiple mechanisms with a high selectivity to gastrointestinal receptors without passing gut-brain barrier,
3. Poor and more local absorption from gastrointestinal tract is important in order to avoid AEs,
4. The main effect with many spasmolytic agents are on the effect of the gastrointestinal motility. In this case, potential therapeutic targets should be identified to change the motility.

In summary, spasmolytic agents represent a mainstay therapy for IBS-related pain and other symptoms such as bloating, abdominal distension, and diarrhea. Overall, most agents demonstrate a favorable safety profile, and some show clinical efficacy. Among these therapies, only Pinaverium, Otilonium, and Peppermint oil are supported by sufficient meta-analytic and high-quality evidence. In contrast, data remain limited for agents such as Alverin, Trimebutin, Cimetropium, and Drotaverin, and are largely insufficient for simeticone or combinations of spasmolytic agents with simeticone. 

In choosing an antispasmodic agent, clinicians should rely on the best available evidence and incorporate it into their routine clinical judgment, rather than basing decisions solely on tradition or personal preference.

### Use of Antispasmodics in Children

Functional gastrointestinal disorders are also common in children. Antispasmodic drugs are used in infantile colic (IC), functional abdominal pain (FAP) and IBS. About 10-30% of infants are reported by caregivers to have prolonged periods of crying known as colic.^124^^,^^125^ Although 35% to 38% of elementary school children report abdominal pain weekly, the prevalence of FAP diagnosed based on Rome criteria is less than 5%. The prevalence of IBS has been reported between 1.2% and 22.6% in different regions and different age groups.^126^^,^^128^

In this section, only studies involving children aged ≤17 years will be considered. There were also some studies evaluating adolescents aged ≥16 years and adult patients together. These studies were also excluded because there were no separate data about children (Table 7).

Mechanisms of action of drugs will not be discussed in detail as they have been mentioned in previous sections. Child-specific AEs will also be mentioned.

## Simeticone

Simeticone is commonly used in the treatment of IC. In a real-word study, 99% of 4004 parents indicated that they used simeticone oral suspension for IC. The most common pattern of use was 5-7 times daily. In 75.7% of the infants, the diagnosis of IC was made by non-healthcare persons such as family member, friend, etc. ^129^

**Clinical efficacy:** In an open-label trial,^130^ the efficacy of simeticone was assessed in 51 healthy infants between 2 and 12 weeks of age with IC. Simeticone drops 0.3 mL (20 mg) were given with each feeding and caregivers reported the infant’s response after 1 day and 1 week of treatment. Symptoms of IC improved or resolved in 78% after 1 day and in 86% after 7 days. The lack of definition of IC and placebo arm were the limitations of the study. In a randomized controlled cross-over trial published in 1988,^93^ 26 infants aged from 1 week to 3 months with IC were enrolled. Infants received one or two droppers of the medication (corresponding to 20–40 mg of simeticon) four times daily, with one dose administered before the evening feeding, for a duration of one week. Subsequently, they were crossed over to the placebo phase without a washout period.Primary outcomes were number of crying episodes a day and severity of crying attack. Simeticone showed statistically significant efficacy, compared to placebo, in reducing the intensity and duration of crying after 4 days of treatment (*P *< .05). They reported that the order of administration did not affect the results of treatment or placebo. Unfortunately, the study did not provide a definition of IC. Moreover, the method of randomization was not clearly described, and there was no information regarding the blinding of parents. In addition, no washout period was implemented.The results for the first study period and the end of the study were not reported separately. Experimental design of these two studies were poor.^93^^,^^130^

In two randomized controlled cross-over studies with specific inclusion criteria of infants with IC, approximately one fourth^92^ to two thirds^131^ of infants improved while receiving simeticone, but this improvement was not better than that observed during placebo treatment. In the study by Danielsson et al,^131^ 32 infants aged from 2 to 8 weeks randomly were administered either simeticone (27.8 mg before each meal) or placebo for 7 days. If the infants were healthy and if the crying was diagnosed as IC, they were included in the study. The placebo and the tested drug had the same smell, taste, colour and texture. Following a 3-day washout period, the administered substances were changed. Five infants were dropped out, unrelated to the study substances. The response was defined as better or much better. The daily duration of crying time, fussing and sleeping, and the numbers of feedings given and stools passed were also recorded. At the end of the study, there were no significant difference between the groups concerning duration, frequency, and intensity of crying, time spent sleeping, number of feedings and stools, irrespective of which treatment was received first.^131^ 67% of the infants improved during treatment, which could be ascribed to a high-grade placebo effect. However, the case definition in that study was highly subjective. Metcalf TJ et al^92^ first randomised 92 infants between 2 and 8 weeks of age with IC, diagnosed based on Wessel criteria,^132^ to either the drug group (0.3 ml with each feeding) or the placebo group for approximately 1 week (range 3 to 10 days) each followed by the alternate substance for the second period. Eighty-three patients completed the study, and 9 infants were excluded from the study for reasons unrelated to drugs. A five-point scale was used to identify the child’s symptoms as; definitely better or symptom free (+2), possibly better (+1), the same (0), possibly worse (-1), or definitely worse (-2). Definite or probable improvement was observed in 25% of the patients in simeticone group and in 29% in placebo group (*P *> .05). The limitations of the study were that there was no washout period and the drugs were not administered for a fixed period. The results for the first study period and the end of the study were not reported separately.

There are also trials using simeticone as a control group. The effect of simeticone in IC has been compared not only with other drugs but also with chiropractic therapy and specialised infant formulas. In an RCT,^133^ 50 infants aged 2 to 10 weeks with IC were randomly assigned to spinal manipulation or simeticone for 2 weeks. Infantil colic was defined as at least 90 minutes crying each day for at least 5 of the 7 previous days. The dose of simeticone was not clear. Parents of all participants were asked to complete an IC diary for the duration of the trial. All 25 in the manipulation group completed the study compared with 16 (64%) in the simeticone group. Four patients in the simeticone group discontinued their medication because they were worse, and they were included in the analysis. It was reported that spinal manipulation significantly reduced crying compared with simeticone (*P* = .04). Because motion palpation is a controversial assessment tool, its use as a reliable indicator of restricted movement in infants younger than 10 week-old is doubtful.^133^ A Cochrane systematic review also considered that the published literature on manipulative therapy, including chiropractic for IC, lacked unbiased information, making it impossible to conclude that they have a positive benefit.^135^

Savino et al^136^ compared an infant formula containing partially hydrolysed whey proteins supplemented with fructooligosaccharides to a standard formula and simeticone (6 mg/kg 2x1/day). The subjects were 267 formula-fed infants aged less than 4 months having IC based on Wessel criteria.^132^ 130 of them randomly assigned to the treatment with the new formula (ST) and 137 to the standard formula and simeticone (CT) group. After a second revision, 20 infants in the ST and 25 in the CT were excluded from the study because they did not satisfy the inclusion criteria. At the end of the study 96 infants in the ST and 103 in the CT completed the study. The parents requested to write down the daily number of colic episodes and crying time for 14 days. After 7 and 14 days, the new formula had a significant decrease in colic episodes compared to standard formula and simeticone group (*P *< .0001). The limitations of the study were its unblinded nature and that 24.5% of the patients randomized did not complete the study. The absence of placebo arm was another limitation for both studies.^134^^,^^136^

In another study, Savino et al.^137^ used simeticon as the control versus L. reuteri. Ninety breastfed colicky infants diagnosed based on Wessel criteria, aged 21-90 days, were randomly assigned either L. reuteri (10^8^ live bacteria per day; 41 infants completed the trial) or simeticone (60 mg/day; 42 infants completed the trial) each day for 28 days. No infants withdrew due to any AEs related to the trial. Primary outcome of the study was a reduction of the daily average of crying time. The secondary outcome was the number of responders versus non-responders in each group at the end of the treatment. Response was defined as a decrease in the daily average crying time out of 50% during the study. Infants receiving L. reuteri showed a significant reduction in daily crying time by day 7 (*P* = .005), On days 14, 21, and 28 crying times were significantly different between the 2 treatment groups (*P* < .001). On day 28, 39 infants (95%) were responders in the probiotic group and 3 infants (7%) in the simeticone group (*P* < .001). No AEs were observed. The lack of a true placebo group in the study was a major limitation that might have affected the outcome. Another limitation of the study was its open-label design. In a recent open-label randomised study conducted by Piatek et al^140^, simeticon was used as control vs a nine-strain bacterial synbiotic (Lactobacillus acidophilus LA-14, Lacticaseibacillus casei R0215, Lacticaseibacillus paracasei Lp-115, Lacticaseibacillus rhamnosus GG, Ligilactobacillus salivarius Ls-33, Bifidobacterium lactis Bl-04, Bifidobacterium bifidum R0071, Bifidobacterium longum R0175 and fructooligosaccharides) in 87 infants with colic (33 were treated with simeticone) fullfilling Wessel’s criteria.^132^ Baseline characteristics of the babies were not different. Responder rates (≥50% reduction from baseline) of the synbiotic group compared to simeticone group were significantly higher for the measures “crying days last 3 weeks” (72% vs 18%, *P* < .0001) and “average evening crying duration last 3 weeks” (85% vs 39%, *P* = .0001), but not for “reduction of average number of crying phases per day in last 3 weeks” (50% vs 42%, *P* > .05). In an another study,^140^ 150 infants who were diagnosed with IC according to Rome III criteria randomly assigned to simeticone (80 mg/day), B.breve CECT7263 (2x10^8^ CFU/day), or a combination of L. fermentum CECT5716 and B. breve CECT7263 (1×10^8^ CFU/day per strain) for 28 days. The main outcomes were duration of crying per day and the percentage of reduction in daily crying from baseline. The daily crying time significantly decreased in all groups. However, babies receiving B.breve had significantly reduction in the duration of crying from baseline comparing to simeticone group. Lack of a true placebo group was the limitation of the both study.

Alves et al^138^ compared the efficacy of PO to simeticone in a crossover double-blind trial in 30 infants, aged 8 to 56 days, with IC diagnosed according to Wessel criteria.^132^ Three of them were excluded and 27 infants were firstly randomized in two groups to receive formulation of leaves of the Mentha piperita (liquid drops; 1 drop per kg body weight) or simeticone (liquid drops; 2.5 mg per kg body weight) daily for 7 days. After the initial 7-day treatment period and a subsequent 3-day washout phase, all participants had their medications switched and were then followed for an additional 7 days. Primary outcomes were number of daily episodes of colic and time spent crying, measured by a chronometer. Primary outcomes were evaluated by mother’s opinion about the responses to the treatment. After 7 days of treatment, all mothers reported decrease of frequency and duration of the episodes of IC and there were no differences between responses to both treatments. There was no AE. Lack of a true placebo group in the study was a major limitation, which might have affected the outcome.

Martinelli M et al^139^ compared a mixture of Matricariae chamomilla L., Melissa officinalis L. and tyndallized Lactobacillus acidophilus (HA122) (Group A) with Lactobacillus reuteri (Group B) and with simeticone (60 mg 2x1/day, Group C). Treatment was given to subjects for 28 days. In this prospective, multicenter, open label, RCT 180 infants with IC, age ≥2 weeks to 4 months, according to Rome II criteria, randomly assigned to these groups (60 patients in each group). One patient of Group B and three patients of Group C were lost at follow-up. Mean duration of crying time (min/day) at the end of treatment (day 28) was the primary outcome of the study. The secondary outcome measure was the number of participants who responded to treatment on day 28. Mean daily crying time at day 28 was significantly lower in Group A (*P* < .001) and Group B (*P *< .001) when compared to Group C. No significant difference was observed between Group A and Group B. At day 28, 68.4% of infants in Group C responded, defined as a decrease in the daily average crying time of 50% from baseline to treatment compared with 95% in Group A and 86.4% in Group B (*P* < .001). No AE was reported in any group. The limitations of the study were being unblinded, open-label design and the lack of placebo.

In a randomized multicenter, controlled, open-label study Raak C et al^142^ compared simeticone with a homeopathic complex medicine (Enterokind) for the treatment of IC. The diagnostic criteria for IC was not given. 125 infants aged ≤6 months were randomly (with an electronically generated block size of 4) assigned to either simeticone (n=51, 1 teaspoon, 3-5 times per day) or Enterokind (n=74, during the acute phase 3 drops every hour, up to a maximum of 6 times per day, the dosage decreased to 3 drops, 3 times a day upon improvement) group. The duration of treatment was 10 days and the response was evaluated according to the Complaint Score (CS), (consists of 9 complaints including sleep disturbances, unmotivated agitation, appetite disturbances, increased crying during and after feeding, regurgitation, vomiting, constipation, loose stool and flatulence, maximum score 17) and Objective Symptoms Score (OSS), maximum score 22, consist of 15 objective symptoms; skin pallor, dry skin, dry mouth, geographic tongue, abdominal bloating, borborygmus, pain on palpation, intestinal spasms on palpation, hepatomegaly, splenomegaly, hard stool, skin rash, intertrigo, coating of the tongue, liquid stool). Although the mean values of both scores decreased at day 10 in both groups, the observed differences in total CS and OSS between day 10 and baseline were higher in Enterokind group. Treatment-related differences were found to be highly significant (*P* < .0001) in favor of Enterokind. Assessments by both the investigators (45.2% vs. 21.3%) and by the patients’ parents (75.3% vs. 21.3%) on day 10 showed that a higher proportion of patients of the homeopathically treated group than in the control group had “no complaints”. In total, one AE in the homeopathically treated group (vomiting, unlikely related to Enterokind) and 4 in the simeticone group (2 allergic rashes, 1 hard stool and 1 viral respiratory tract infection. One of allergic reactions was probably related to simeticone). The limitations of the study were unequal distribution of the cases between the groups, its open-label design, the use of non-validated scales for the assessment of complaints and symptoms, high number of dropped-out patients (20 total).

**Safety:** simeticon may prevent the effect of thyroxine in infants with hypothyroidism; however, this observation is based on a single case report. Likewise, in premature infants, it has been reported to cause retinopathy, but this adverse effect was also described in only one case report.^143^^,^^144^

**Conclusion:** There was substantial heterogeneity among the studies regarding diagnostic criteria, dosage and duration of treatment, methods of randomization, outcome measures, and overall methodological quality. The available evidence does not provide conclusive support for the efficacy of simeticon in the treatment of infantile colic. (Grade D).

### Peppermint oil

**Clinical efficacy:** Data regarding the efficacy of PO in children is limited. In a 2-week double-blind RCT,^145^ 50 children aged 8 years and older with IBS, who met the Manning or Rome criteria were randomized to receive either enteric-coated PO capsules (3*×*1/day (≤45 kg) to 3*×*2/day (>45 kg); depending on the weight, each capsule consisted of 187 mg PO) or placebo (arachis oil). Eight (16%) children withdrew from the study due to non-drug related reasons, and 42 children (21 per group) completed the study. Outcome was assessed using the 15-item Gastrointestinal Symptom Rating Scale (GSRS, measures frequency, duration and impact on daily life) and scales recording severity of symptoms and change in symptoms. Outcome was based on parent and child report. Improvement in the severity of GSRS was seen in 76% of children receiving PO vs 19% of children receiving placebo (*P *< .001). Improvements in the change of GSRS were observed in 71.4% of the patients in intervention group compared with 42.9% receiving placebo (1-sided *P* = .059). The GSRS showed no significant differences between groups. Although it was not reported in the study, OR for improvement was 3.33 (95%CI 0.93 to 12.01). Symptoms worsened in 6 (29%) patients receiving placebo compared to 0% in intervention group (*P *< .05). No AEs were reported by either the investigator or the patients. Absence of detail on randomization, the lack of the method of analysis of the daily diaries were the limitations of the study.

In another study,^146^ the efficacy of PO (187 mg 3*×*1/day, 2*×*1/day for children <45 kg) in children with FAP, functional dyspepsia or IBS, diagnosed based on Rome III criteria, was compared to symbiotic (Bacillus coagulans + fructooligosaccharids) and placebo (1 mg folic acid). The study was the placebo-controlled, randomized but single-blinded (only nurse who has completed questionnaire was unaware of the protocol). Primary outcomes were decreasing in duration, severity and frequency of FAP. Eighty-eight (73.3%) out of 120 enrolled patients (PO 34, symbiotic group 29 and placebo group 25 patients), aged 4-13 years, completed the 1-month trial. None of the patients withdrew from the trial due to AEs. Analyses showed that improvement in pain duration, frequency and severity in the PO group was better than the placebo group (*P* = .0001, *P* = .0001 and *P* = .001, respectively). Peppermint oil was superior to symbiotic in decreasing pain duration and severity (*P* = .040 and *P* = .013, respectively). No known AE or intolerance was seen with PO or symbiotic groups. There were 3 important limitations of the trial; heterogeneity of the diagnosis, lack of ITT analysis (26.7% of the patients did not complete the trial), and single-blinded nature.

**Safety:** Peppermint oil may cause bronchospasm, respiratory depression and apnea in infants and young children. It can cause jaundice in newborns, particularly in those with glucose-6-phosphate dehydrogenase deficiency. Rectal burning, esophageal pain or heartburn and allergic reactions are the other reported AEs.^147^

**Conclusion:** Further well designed researches with larger number of participants and are needed (Grade C).

### Mebeverine Hydrochloride

**Clinical efficacy:** To date, only one study has evaluated the effect of MO in the pediatric population. In an RCT^149^ on 115 children aged 6-18 years having FAP according to Rome III criteria were randomized to receive Mebeverine Hydrochloride (MH) (n=59, 135 mg, 2*×*1/day) or placebo (n=56) for 4 weeks and were followed for 12 weeks. A total of 87 (44 in MH group) patients and 79 (40 in MH group) patients completed the 4-week and 12-week follow-up. Three subjects in MH group discontinued MH due to AEs (2 had drowsiness and nervousness, and 1 nausea), and 1 patient in placebo group due to antibiotic use. The remaining patients did not continue the study for reasons unrelated to AEs. Response was defined as ≥2 points reduction in the 6-point pain scale or “no pain”. Per-protocol (PP) and intention-to-treat (ITT) analysis were conducted. The treatment response rates in the MH and placebo groups based on the PP and ITT analyses were 54.5% (PP) and 40.6% (ITT) versus 39.5% (PP) and 30.3% (ITT) at week 4 (*P* > 0.05), and 72.7% (PP) and 54.2% (ITT) versus 53.4% (PP) and 41.0% (ITT) at week 12 (*P* > 0.05), respectively. 

The major limitation of the study was the drop-out rate of 24.3% at the 4th week and 31.3% at the 12th week.

**Safety:** Dry mouth experience was significantly more common in MH group compared to placebo group (43.1% vs 23.2%, *P* = .047) in abovementioned RCT. There was no difference between the two groups regarding other AEs, including insomnia, nausea, drowsiness, vomiting, fatigue, headache, dizziness, and loss of appetite.

Conclusion: Mebeverine hydrochloride is not recommended in pediatric patients with FAP (Grade D).

### Drotaverin Hydrochloride

**Clinical efficacy:** Research on the effect of DRH in the pediatric population remains scarce, with only one study conducted to date. In an RCT consisting 132 children (age 4-12 years) with RAP randomized to receive DRH (n=66, 20 mg as syrup for children between 4-6 years of age, 40 mg tablet for older children) or placebo (in the same formats) 2*×*1/day for 4 weeks.^150^ Sixty-four patients in the DRH group and 60 patients in the placebo group completed the study. RAP was defined as at least three episodes of pain interfering with normal activities within a 3-month period (Apley criteria).^151^ Primary outcome measures were number of episodes of pain during 4 weeks of use of drug/placebo and number of pain-free days. Number of school days missed during the study period, parental satisfaction, and occurrence of solicited AEs were secondary outcome measures. Although the mean pain-free days were not different between groups (*P* = .23), the mean number of pain episodes (*P* = .015) was statistically better and the main percentage of school absence tended to be more improved (*P* = .054) in the treatment group. Significant improvement in parental satisfaction score was noticed (*P *< .05). The main limitations of the study were that RAP definition was made based on Apley’s criteria and short follow-up period.

In the abovementioned study, the frequency of AEs during the follow-up period was comparable between children receiving DRH and those receiving placebo (46.9% vs 46.7%; *P* = .98). Fever, cough, and vomiting, not causally related to the study drug, were the most common AEs in both groups (*P *> .05). A macular rash was observed in four patients in the DRH group and one in the placebo group. One patient developed urticerial rash which required discontinuation of the therapy.

**Conclusion:** The efficacy of DRH is not established in children because of the insufficient data.

### Hyoscine and Its Derivatives

Although IC usually resolves spontaneously by the fourth month, drugs with antispasmodic activity are commonly used in the treatment of IC because of the assumption that intestinal smooth muscle spasms cause colic.^145^

The most commonly used hyoscine derivatives are hyoscine butylbromide (N-butylscopolamine), primarily employed for gastrointestinal and biliary tract spasms; hyoscine hydrobromide (scopolamine hydrobromide), mainly used for the prevention of motion sickness and nausea; and methscopolamine bromide, which is utilized for the management of functional gastrointestinal disorders.

**Clinical efficacy:** Methyl scopolamine has been used in the treatment of IC since the 1940s. Jurop administered scopolamine (0.05 mg to 0.15 mg, 5-6/day) to 61 with IC. He reported that pain improved in 54 cases.^152^ In a non-randomized open-label case series Wessel evaluated the effect of scopolamine in 13 infants with paroxysmal fussing and obtained excellent response in 9 cases.^134^ The drug dose and duration of administration were adjusted according to the patient’s response.

The first RCT conducted of methylscopalamine in IC was published in 1955.^153^ The trial included 40 consecutive babies (mean age 4.5 weeks) with “3 months” colic. The patients were randomly assigned to either the drug (0.05 mg scopolamine 4*×*1/day sublingually before feeds) or the placebo (ascorbic acid solution) group. The average duration of treatment was 17.9 days. The response to treatment was evaluated with a scale ranging from +4 to -4 (+4 completely recovered, -4 much worse). Improvement was seen in 70% of the infants in the treatment group and in 80% in the placebo group. In the treatment group 25% of the parents reported to be worse or much worse after treatment, as compared with none of the infants in the placebo group. No data was given about AEs. The main limitations of the study were unclear diagnostic criteria and indefinite treatment duration.

Homatropine methylbromide is a quaternary ammonium muscarinic acetylcholine receptor antagonist belonging to the group of medicines called anti-muscarinics. Its structure is similar to hyoscyamine. In an article published in 1979,^154^ 110 babies aged between 2 and 6 weeks were randomly divided into 4 groups. The trial was double-blind. Infantile colic was defined as at least 90 minutes crying each day for a minimum of 6 of the preceding 7 days. The first group (n=27) received 10 mg phenobarbital plus 0.25 mg homatropine methylbromide in 20% alcohol per 2.5 mL, the second group (n=29) received 10 mg phenobarbital in 20% alcohol per 2.5 mL, the third group (n=28) received 20% alcohol, and the fourth group received placebo (n=26, water and artificial coloring). A dosage of 2.5 ml 3*×*1/day was prescribed initially for all participants. Each child was seen weekly for 2 weeks. The child’s response was graded a purely subjective scale ranging from -2 (crying increased by more than 90 min/day) to +2 (complete cessation of crying). 13 children (1, 2, 3, and 7 children in Groups 1, 2, 3, and 4, respectively) didn’t complete the study. After two weeks, more than 70% of all patients had clinical improvement, mostly in the placebo group (84%). No data was given AEs. Given the inclusion of substances such as alcohol and phenobarbital—agents now recognized as inappropriate and potentially harmful for infants—these findings must be interpreted with caution, as the study design itself carries safety concerns that would render such an intervention unacceptable in contemporary pediatric practice. 

**Safety:** Long-term use of hyoscyamine in children may cause dry mouth, urine retention, blurred vision, tachycardia, drowsiness and constipation.^148^ Scopolamine intoxication may cause delirium characterized by deteriorated cognitive function, changed mental status, psychotic behaviors including bizarre actions, hallucinations, aggressive behavior, hyperactivity and inherent speech.^155^

**Conclusion:** The efficacy of hyoscyamine in the treatment of IC is not established (Grade D). Due to its numerous adverse effects and potential drug–drug interactions, its use in children is generally discouraged.

### Dicyclomine Hydrochloride

Although it has been shown to be effective for IC, its use in infants <6 months old is contraindicated because of severe AEs.^153^^,156^ Dicyclomine should not be used during lactation. Therefore, its use with this indication will not be discussed.

### Cimetropium Bromide

**Clinical efficacy:** Cimetropium bromide, a semisynthetic derivative of scopolamine, is one of the medicines used to treat IC. In a study from Italy^126^ including 97 infants aged 15 to 60 days with IC diagnosed based on Wessel criteria,^132^ each infant was randomly assigned to either CB (1.2 mg/kg at the onset of each crisis) or placebo (a solution with the same color, smell, taste, and package) for 3 days was compared to placebo. The parents were requested to record the duration of crying and AEs (meteorism, vomiting, sleepiness, restlessness, inappetence, cutaneous reactions, constipation, respiratory distress, apnea). The therapy was considered efficacious if crying ended within 15 minutes after administration of the compounds. Fourty-three infants in each group completed the study. Although the average number of crises per day was not different between groups, CB decreased the average duration of crying for each crisis significantly compared to placebo (average time of crying 17.3 ±12.6 vs 47.5 ± 28.5 minutes, respectively, *P* < .005). The response to CB, defined as cessation of crying within 15 minutes after administration of the compound, was 74%, compared with 33% in the placebo group. (*P* = .0001). Cimetropium bromide was associated with increased sleepiness compared to placebo. However, the baseline characteristics of the patients and the total daily crying time were not reported, and no details were provided regarding the method of randomization.

In another study,^^157^^ 2 different doses of CB were randomly compared in 40 patients with infant colic of an average age 4.4 weeks (range not reported) based on Wessel criteria.^132^ One group (n=20) received 1.2 mg/kg cimetropium and the other group (n=20) 2.0 mg/kg 1 hour before bottle. Infants received the first treatment for 7 days, then crossed to the other treatment group for the next seven days. Reduction in the number of crying episodes and in their duration was recorded. The duration of crying decreased in both groups, with a greater reduction observed in the higher-dose group, whereas the frequency of crying episodes decreased in both groups, favoring the lower-dose group. Hovewer, the difference was not significant. There was no difference between arms regarding the percentage of infants with excellent or good improvement in the symptoms (85% of cases in both groups). The two groups of infants differed at baseline. There was no information on the method of random sequence, blinding of parents and outcome measures. Four infants receiving the higher dosage had constipation.

**Conclusion:** Compared to placebo, CB may be beneficial in reducing crying time or relieving other symptoms of IC, but the quality of evidence is low (Grade C).

### Trimebutine Maleate

**Clinical efficacy:** In a study involving 78 children (selected out of a total 345 children aged 4-18 years,) with IBS diagnosed based on the Rome III criteria, half of the randomly selected patients treated with TM (3 mg/kg/day, 3x1/day) for 3 weeks and while the remaining 39 patients did not take any medication.^128^ Clinical recovery defined as “adequate relief” was seen in 94.9% of the TM group versus in 20.5% in non-medicated group (*P *< .001). There was no information about AEs. Absence of placebo is the limitation of the study.

*Conclusion:* There are not enough studies to recommend its use (Grade D).

#### Acknowledgements:

The authors would like to acknowledge Merve Evren, PhD, for her contribution to the preparation of the figures, which reflect both technical precision and artistic quality.

## Figures and Tables

**Table 1.** Comparison of Meta-analyzes by Their Analyzed Studies and Type of Drugs

	Jailwala 2000	Poynard 2001	Lesbros 2004	Ford 2008	Enck 2010	Ruepert 2011	Martínez 2012	Black 2020	Rueperrt 2013	Annahazi 2014	Ford 2018
**Alverine** Mitchell 2002 Wittmann 2010					**(+)**	**(+)**	**(+)** **(+)**	**(+)**	**(+)**	**(+)** **(+)**	**(+)**
**Butylscopamin**						**(+)**					
**Cimetropium** Piai 1987 Centonze 1988 Pasaretti 1989 Dobrilla 1990 Piai Magazza 1979	**(+)** **(+)** **(+)**	**(+)** **(+)** **(+)** **(+)**	**(+)** **(+)** **(+)** **(+)**	**(+)** **(+)** **(+)**	**(+)** **(+)** **(+)**	**(+)** **(+)** **(+)**		**(+)** **(+)**	**(+)** **(+)** **(+)**		**(+)** **(+)** **(+)** **(+)**
**Dicyclomine** Page 1981	**(+)**			**(+)**	**(+)**	**(+)**	**(+)**		**(+)**		**(+)**
**Drotaverine** Misra and Pandey 2000 Rai 2014								**(+)** **(+)**			**(+)** **(+)**
**Hyoscine** Ritchie 1979 Nigam 1984 Schaefer 1990		**(+)** **(+)** **(+)**	**(+)** **(+)** **(+)**	**(+)** **(+)** **(+)**	**(+)** **(+)** **(+)**		**(+)** **(+)** **(+)**	**(+)**	**(+)** **(+)** **(+)**		**(+)** **(+)** **(+)**
**Mebeverine** Connell 1965 Tasman-Jones 1973 Tasman-Jones 1976 Berthelot 1981 Secco 1983 Kruis 1986 Enck 2005	**(+)** **(+)** **(+)**	**(+)** **(+)** **(+)** **(+)** **(+)**	**(+)** **(+)** **(+)** **(+)**	**(+)**	**(+)**		**(+)** **(+)** **(+)** **(+)** **(+)** **(+)**	**(+)**	**(+)**	**(+)** **(+)** **(+)**	**(+)**
**Otilonium** dArienzo 1980 Barbier 1981 Baldi 1983 Baldi 1991 Castiglione 1991 Battaglia 1998 Glende 2002 Clave 2011	**(+)** **(+)**	**(+)** **(+)** **(+)** **(+)**	**(+)** **(+)** **(+)** **(+)**	**(+)** **(+)** **(+)** **(+)**	**(+)** **(+)** **(+)**	**(+)** **(+)** **(+)**	**(+)** **(+)** **(+)** **(+)** **(+)**	**(+)** **(+)** **(+)** **(+)**	**(+)** **(+)** **(+)** **(+)**	**(+)** **(+)** **(+)**	**(+)** **(+)** **(+)**
**Pargeverine** Pulpeiro 2000								**(+)**			**(+)**
**Peppermint oil** Rees 1979 Dew1984 Nash 1986 Lech 1988 Carling 1989 Czalbert 1990 Liu 1997 Capanni 2005 Cappello 2007 Merat 2010 Cash 2016 Mosaffa-Jahromi 2016 Weerts 2019	**(+)**		**(+)** **(+)** **(+)** **(+)** **(+)**		**(+)** **(+)** **(+)** **(+)**	**(+)** **(+)** **(+)** **(+)** **(+)**		**(+)** **(+)** **(+)** **(+)** **(+)** **(+)** **(+)** **(+)** **(+)** **(+)** **(+)** **(+)** **(+)**	**(+)** **(+)** **(+)** **(+)** **(+)**		**(+)**
**Pinaverium** Levy 1977 Dubarry 1977 Delmont 1981 Virat 1987 Awad 1995 Chen 2004 Zheng 2015	**(+)**	**(+)** **(+)**	**(+)** **(+)**	**(+)** **(+)** **(+)**	**(+)** **(+)** **(+)**	**(+)** **(+)** **(+)** **(+)** **(+)** **(+)**	**(+)** **(+)**	**(+)** **(+)**	**(+)** **(+)** **(+)** **(+)** **(+)** **(+)**	**(+)**	**(+)** **(+)** **(+)** **(+)**
**Pinaverium/simeticone** Remes-Troche 2011							**(+)**				
**Pirenzipine** Givarry 1989	**(+)**			**(+)**	**(+)**	**(+)**	**(+)**		**(+)**		**(+)**
**Prifinium** Piai 1979	**(+)**		**(+)**	**(+)**	**(+)**	**(+)**			**(+)**		
**Propinox** Pulperio 2000				**(+)**	**(+)**	**(+)**			**(+)**		
**Rociverine** Ghidini 1986	**(+)**					**(+)**			**(+)**		**(+)**
**Trimebutine** Luttecke 1975 Luttecke 1978 Moshal 1979 Fielding 1980 Luttecke 1980 Ghidini 1986	**(+)** **(+)**	**(+)** **(+)** **(+)** **(+)**	**(+)** **(+)**	**(+)** **(+)** **(+)**	**(+)** **(+)** **(+)**	**(+)** **(+)** **(+)**	**(+)** **(+)** **(+)** **(+)**	**(+)** **(+)** **(+)**	**(+)** **(+)** **(+)**		**(+)** **(+)**

**Table 2.** Comparison of Meta-analyses According to the Drugs They Evaluate

	Jailwala 2000	Poynard 2001	Lesbros 2004	Ford 2008	Enck 2010	Ruepert 2011	Martínez 2012	Black 2020	Ford 2018
Number of studies	16	21	24	23	22	22	18	26	26
Number of good studies	7		12	12	12				
Alverine				(-)		(-)	(-)		(-)
Butylscopamin									
Cimetropium	(+)^#^	(+)	(+)	(+)		(+)			(+)
Dicyclomine				(+)		(+)	(-)		(+)
Hyoscine		(+)	(+)	(+)			(+)		(+)
Mebeverine		(+)	(+)	(-)		(-)	(-)		(-)
Otilonium	(+)^#^	(+)	(+)^*^	(+)		(+)	(+)		(+)
Pargeverine									
Peppermint oil			(+)			(+)			(+)
Pinaverium	(+)^#^	(-)	(-)	(+)		(+)	(-)		(+)
Pinaverium/simeticone							(-)		
Pirenzipine				(-)		(-)			(-)
Prifinium				(-)					(-)
Propinox				(-)					(-)
Rociverine				(-)		(-)			
Trimebutine	(+)^#^	(+)	(-)	(-)		(-)	(-)		(-)
Efficacy (OR, CI 95%)		2.13 (1.77-2.58)	1.5 (1.2-1.9)	0.68 (0.57-0.81)	1.97 (1.59-2.45)	1.49 (1.25-1.77)	1.55 (1.33-1.83)	0.64 (0.49-0.84)	0.65 (0.56-0.76)
NNT	1.6-6.7			5	5-6		10 (6-41)		5 (4-8)

^#^No specific data provided; *Positive, if low quality studies excluded, (+): Better than placebo, (-): Comparable with placebo.

NNT, number needed to treat.

**Table 3.** Total Number of Articles*

Adult Population Studies															
	Alverine	Cimetropium	Dicyclomine	Drotaverine	Hyoscine	Mebeverine	Otilonium	Peppermint oil	Pinaverium	Prifinium	Propinox	Rociverine	Trimebutine	Simeticone	Antispasmodics
Total number of articles in Pubmed	10	15	56	13	87	57	44	109	39	9	1	3	81	121	1610
Clinical Trials	5	10	12	4	18	26	10	21	17	3	0	2	19	66	316
Meta-analysis	1	2	3	1	4	6	2	8	6	0	0	1	8	5	32
RCT	4	9	8	3	15	24	9	18	13	3	1	1	12	56	201
Review	2	1	8	1	10	10	10	53	9	0	0	0	10	14	347
Systematic Reviews	1	0	3	1	2	4	2	15	4	0	0	0	4	5	36
Pediatric Population Studies															
Total number of articles in Pubmed	1	0	17	0	12	26	0	15	8	0	0	0	12	26	0
Clinical Trials	1	0	2	0	5	19	0	4	8	0	0	0	5	17	0
Meta-analysis	0	0	0	0	0	1	0	2	0	0	0	0	1	1	0
RCT	1	0	1	0	3	18	0	4	7	0	1	0	5	15	0
Review Articles	0	0	3	0	0	0	0	8	0	0	0	0	1	4	0
Systematic Reviews	0	0	0	0	0	0	0	5	0	0	0	0	0	0	0

*(Butylscopalamine or dicyclomine or pinaverium or mebeverine or peppermint oil or otilonium or alverine or trimebutine or cimetropium or simeticone or drotaverine or rociverine or prifinium or propinox) and (functional gastrointestinal disorders or irritable bowel syndrome or functional constipation or dyspepsia).

RCT, Randomized controlled trials.

**Table 4.** Evidence Quality Assessment and Strength-of-Evidence Recommendation

Grade	Quality of Evidence	Definition
A	High	Further evaluation is unnecessary to change our confidence in the estimate of effect. The statement can be supported by several high quality studies with consistent results or in special cases by one large, high-quality multicenter trial.
B	Moderate	Further research is likely to have an important effect on our confidence in the estimate of effect and might change the estimate. The statement can be supported by one high-quality study or several studies with some limitations.
C	Low	Further research is very likely to have an important effect on our confidence in the estimate of effect and is likely to change the estimate. The statement can be supported by one or more studies with severe limitations.
D	Very low	Any estimate of effect is very uncertain. The statement can be supported by expert opinion or one or more studies with very severe limitations, or there might be no direct research evidence.

RCT, Randomized controlled trials.

**Table 5.** Publications for Antispasmodic Drugs in IBS

Author, year	Intervention, duration	Participants	Diagnosis/ criteria	Study design	Primary outcome measures	Results	Limitations	Adverse events
**Hyoscine butylbromide**								
Ritchie et al. 1979^17^	Hyoscine butylbromide 4 × 10 mg/day, 12 weeks	Total 96 patients making up 12 randomised blocks	IBS/NA	RCT	Global symptom improvement	Similar with placebo	Diagnostic criteria not defined	NA
Khalif et al. 2009^18^	Hyoscine butylbromide 3 × 20 mg/day, 2 weeks	45 patients placebo, 37 patients Hyoscine butylbromide	IBS, ROME II	Placebo controlled non-randomzed study	Abdominal pain relief, VAS score	Significant improvement IBS-D group	Not randomized	No side effects were reported
Mueller-Lissner et al., 2006^20^	Hyoscine butylbromide 3 × 10 mg/day, 3 weeks	400 patients Hyoscine butylbromide, 390 patients paracetamol, 394 patients placebo, 387 patients Hyoscine butylbromide+paracetamol	Abdominal pain	RCT	Abdominal painrelief, VAS score	Significant abdominal pain improvement	No spesific group was defined’	Hyoscine butylbromide 16%, vs. placebo 11%
Lacy et al. 2013^19^	Hyoscine butylbromide 20–100 mg, on demand 2 episode after 4 week run in period	88 patients Hyoscine butylbromide, 87 patients placebo	Abdominal pain associated with cramping	RCT	Abdominal pain improvement	Significant reduction for abdominal pain	No spesific group was defined	Hyoscine butylbromide 10.2% vs. placebo 10.3%
**Cimetropium bromide**								
Piai et al, 1987^25^	Cimetropirum bromide 3 x 50 mg/day, 12 weeks	15 patients Cimetroprium bromide, 15 patients placebo	IBS/NA	RCT	Abdominal pain	Significantly improved	-	2 events in Cimetropium bromide group vs. one event in placebo group
Ferrari et al, 1986^26^	Cimetropium bromide 2 × 50 mg/day, 6 weeks	20 patients Cimetropium bromide, 20 patients Otilonium bromide	IBS, excluded organic disease	Comparative study	Abdominal pain and global symptom relief	Significant decreases for abdominal pain and global symptoms	Not placebo controlled	No side effects were reported
Centonze et al., 1988^27^	Cimetropium bromide 3 × 50 mg/day, 24 weeks	23 patients Cimetroprium bromide, 21 patients placebo	IBS/NA	RCT	Abdominal pain	Significant improvement	-	Cimetropium bromide 48% vs. 29% placebo
Passaretti et al. 1989^28^	Cimetropium bromide 3 × 50 mg/day, 4 weeks	10 patients Cimetroprium bromide, 10 patients placebo	IBS/NA	RCT	Global clinical condition	Significant improvement in IBS-C group	Not designed for specific symptom	Similar with placebo
Dobrilla et al. 1990^29^	Cimetropium bromide 3 × 50 mg/day, 12 weeks	35 patients Cimetroprium bromide, 35 patients placebo	IBS/NA	RCT	Abdominal pain	Significant improvement	-	6 events in Cimetropium bromide group vs. 1 event in placebo group
Peppermint oil								
Rees et al. 1979^43^	Peppermint-oil 0.2 ml, 2 capsules/day, 3 weeks	16 patients	IBS/NA	Placebo controlled cross-over study	Abdominal symptoms	Significant improvement	Not randomised and lacking of diagnostic criteria of IBS	Heartburn in 2 patients receiving Peppermint-oil
Dew et al. 1984^44^	Peppermint-oil 0.2 ml, 3-6 capsules/day, 2 weeks	29 patients	IBS/NA	Placebo controlled cross-over study	Abdominal symptoms	Significant improvement	Not randomised and lacking of diagnostic criteria of IBS	NA
Nash et al. 1986^45^	Peppermint-oil 0.2 ml, 3 × 2 capsules/day, 2 weeks	41 patients (33 completed)	IBS/NA	Placebo controlled cross-over study	Abdominal pain	Comparable with placebo	-	Two patients discontinued therapy because of nause and vomiting due to peppermint-oil, heartburn in 6 patients receiving Peppermint-oil
Liu et al. 1997^46^	EC peppermint-oil 3 × 187 mg/day, 4 weeks	52 patients EC peppermint-oil, 49 patients placebo	IBS/NA	RCT	Abdominal pain, other symptoms	Significant improvement for abdominal pain and abdominal distension	-	Similarwith placebo
Cappello et al. 2007^47^	Peppermint oil 2 × 225 mg/day, 4 weeks	24 patients Peppermint-oil, 26 patients placebo	IBS, ROME II	RCT	IBS sypmtoms	Significant improvement	-	One patient stopped Peppermint-oil drug (heartburn, minty taste)
Merat et al. 2010^48^	Peppermint-oil 3 × 187 mg/day, 8 weeks	33 patients Peppermint-oil, 27 patients placebo	IBS, ROME II	RCT	Abdominal pain	Significant improvement	High loss rate of participants	Similar with placebo
Cash et al. 2016^40^	Peppermint-oil 3 × 180 mg/day, 4 weeks	35 patients Peppermint-oil, 37 patients placebo	IBS, ROME III	RCT	IBS symptoms	Significant improvement	-	Similar with placebo
Mosaffa-Jahromi et al. 2016^49^	AnisEncap, and Colpermin 3 × 187 mg/day, 4 weeks	39 patients AnisEncap, 38 patients Colpermin, 39 patients placebo	IBS, ROME III	RCT	IBS symptoms	Significant improvement with AnisEncap (75%-ITT) and Colpermin (52.5%-ITT)	-	Cimetropium bromide 48% vs. placebo 29% vs. Colpermin 26.31%, 15.15% withdrawn in Colpermin group
Weerts et al. 2020^50^	EC peppermint-oil 3 × 182 mg/day, ileocolonic-release peppermint-oil 3 × 182 mg/day, 8 weeks.	62 patients EC peppermint-oil, 63 ileocolonic release peppermint-oil, 64 patients placebo	IBS, Rome IV	RCT	Abdominal pain	NS	-	4.8% discontinued therapy in EC peppermint-oil group, 7.9% discontinued therapy in ileocolonic release peppermint-oil group, 1.6% discontinued therapy in placebo group (*P *< .005)
Nee et al. 2021^51^	EC peppermint-oil capsules 3 × 180 mg/day, 6 weeks	46 patients EC peppermint-oil, 87 patients placebo	IBS, Rome IV	RCT	IBS Severity Scoring System	NS	Predominanty white, middle-aged women with IBS	34.8% discontinued therapy in EC peppermint-oil group and 18.4% patients withdrew study (, including 9.2% in placebo group because of AEs.
**Otilonium bromide**								
Villagrasa et al 1991^73^	Otilonium bromide 3 × 40mg/day, high content roughage, 114 weeks	61 patients Otilonium bromide, 53 patients placebo	IBS-Clinical	Open-label, randomized, parallel-group	Abdominal pain, abdominal distension, bowel movements	Significant improvement in abdominal pain, abdominal distension, bowel movements	Not placebo controlled	No side effects were reported
Baldi F 1991^74^	Otilonium bromide 3 × 40mg/day, placebo, 4 weeks	72 patients	IBS-Clinical	RCT	Abdominal pain, bloating, bowel movements	Significant improvement in abdominal pain, bloating, no difference in bowel movements	Small sample size, short study duration	One mild nausea
Battaglia G 1998^75^	Otilonium bromide 3 × 40mg/day, 15 weeks	157 patients Otilonium bromide, 160 patients placebo	IBS, Drossman’s diagnostic criteria	RCT	Abdominal pain, abdominal distension and disturbed defecation	Significant improvement in abdominal pain, global positive assessment, abdominal distension, and VAS, no difference between Otilonium bromide and placebo group in bowel movements	High placebo effect	One prostate disturbance and one dizziness with Otilonium bromide
Clave’ P, 2011^65^	Otilonium bromide 3 × 40mg/day,placebo, 15 weeks	356 patients	IBS, Rome II	RCT	Change in the frequency of abdominal pain	Significant improvement in the frequency of abdominal pain, abdominal bloating, IBS global symptoms, and stool frequency. No differences in pain intensity, mucus in stool and consistency of stools	Only patients with longer-standing, stable IBS symptoms included, only one primary endpoint	Two dry mouth and one nausea with Otilonium bromide
Chmielewska-Wilkoń D, 2014^79^	Otilonium bromide 3 × 20mg/day, Otilonium bromide 3 × 40mg/day, Otilonium bromide 3 × 80mg/day,, 4 weeks	24 patients Otilonium bromide 3 × 20 mg/day, 23 patients Otilonium bromide 3 × 40 mg/day, 23 patients Otilonium bromide 3 × 80 mg/day, 23 patients placebo	IBS, Rome II	RCT parallel group study	Abdominal discomfort, intestinal habits, number of daily evacuations and stool consistency	Significant improvement in intensity and frequency of abdominal discomfort, bloating and pain with Otilonium bromide treatment. Global discomfort index revealed significant improvement among increasing Otilonium bromide doses.	Small sample size, short study duration, strong placebo effect and variation in patient characteristics	One headache, one dry mouth and one nausea with Otilonium bromide.
**Trimebutine maleate**								
Luttecke K, 1978^^,^83^	Trimebutine maleate 3 × 200 mg/day, placebo, 3 days	39 patients	IBS, Clinical	Double-blind crossover, placebo controlled-randomized	Patients preference of treatment according the effect	Patients preference favour of Trimebutine maleate	Small sample size, very short study duration	One vomiting, 6 mild tiredness and 6 mild hot and cold sensation with Trimebutine maleate
Luttecke K, 1980^84^	Trial 1: Trimebutine maleate 3 × 200 mg/day vs. placebo, 3 days Trial 2: Trimebutine maleate 3 × 100 mg/day vs. placebo, 3 days Trial 3: Trimebutine maleate 3 × 200 mg/day vs. Mebeverine hydrochloride 4 x 100 mg/day, 2 weeks	Trial 1, 2: 45 patients Trial 3: 40 patients	IBS, Clinical	Trials 1 & 2: RCT cross-over (3 days) Trial 3: Double dummy, cross-over	Trial 1, 2, 3: Patients preference of treatment according the effect	Trial 1: Patients preference favour of Trimebutine maleate Trial 2: Patients preference was not different between the two groups Trial 3: Patients preference was not different between the two groups	Small sample size, very short study duration	Trial 1 and 2: One vomiting, 7 mild tiredness and 6 mild hot and cold sensation in Trimebutine maleate groups Trial 3: AEs reported were transient No SAEs reported
Moshal MG, 1979^85^	Trimebutine maleate 3 × 200 mg/day, placebo, 4 weeks	20 patients (median 27 years)	IBS, Clinical	RCT, cross-over	Constipation, abdominal pain	Significant improvement in abdominal pain and constipation with both Trimebutine maleate and placebo after 4 weeks, but only with Trimebutine maleate after 8 weeks	Small sample size, short study duration	No side effects were reported
Fielding et al, 1980^86^	Trimebutine maleate 3 × 200 mg/day & highfibre diet vs. placebo & highfibre diet, 24 weeks	60 patients	IBS-Clinical	RCT, parallel- group	Abdominal pain, bowel habits, abnormally palpabl or tender of colon	Abdominal pain bowel habits, abnormally palpable or tender of colon	NS	NA
Piai G et al, 1979^88^	Prifinium bromide 3 × 30 mg/day, placebo, 3 weeks.	18 patients	IBS, Clinical	RCT, cross-over study	Global treatment response (abdominal pain, flatulence, constipation, and/or diarrhea)	Significant improvement	Short study duration, small sample size	Mouth dryness and blurred vision, but rare
**Dicyclomine hydrochloride**								
Page et al, 1981^91^	Dicyclomine hydrochloride 4 × 40 mg/day,., 2 weeks	48 patients Dicyclomine hydrochloride, 49 patients placebo	IBS, Clinical	RCT, parallel-group	Physician’s global assessments, patient’s self assessments of treatment and duration of abdominal pain	Significant improvement	Short study duration	NA
**Pinaverium bromide**								
Levy 1977^56^	Pinaverium bromide 3 × 50 mg/day, 2 weeks	22 patients Pinaverium bromide, 22 patients placebo	IBS, Clinical	RCT	OSR, API, ADI, TPN	Favours Pinaverium bromide	NA
NA
Delmont 1981^121^	Pinaverium bromide 3 × 50 mg/day, 4 weeks	30 patients Pinaverium bromide, 30 patients placebo	IBS, Clinical	RCT	OSR, API, ADI, TPN	Favours Pinaverium bromide	NA	NA
Virat 1987^57^	Pinaverium bromide 3 × 50 mg/day, 4 weeks	30 patients Pinaverium bromide, 30 patients placebo	IBS, Clinical	RCT	OSR, API, ADI, SFI	Favours Pinaverium bromide	NA	NA
Awad 1995^63^	Pinaverium bromide 3 × 50 mg/day, 3 weeks	20 patients Pinaverium bromide, 20 patients placebo	IBS, Rome I	RCT	OSR, API, ADI, SFI SCI, ASI	NS	NA	NA
Zhao 2004^122^	Pinaverium bromide 3 × 50 mg/day, 4 weeks	30 patients Pinaverium bromide, 30 patients placebo	IBS, Rome II	RCT	OSR, APR, ADI, TPN	Favours Pinaverium bromide	NA	NA
Zhang 2011^123^	Pinaverium bromide 3 × 50 mg/day, 4 weeks	18 patients Pinaverium bromide, 10 patients placebo	IBS, Rome III	RCT	OSR, API, ADI, SFI, ASI	NS	NA	NA
Zheng 2015^61^	Pinaverium bromide 3 × 50 mg/day, 4 weeks	218 patients Pinaverium bromide, 209 patients placebo	IBS, Rome III	RCT	OSR, APR, ADI, SFI, SCI	Favours Pinaverium bromide	NA	NA
**Simeticone**								
Wittmann et al 2010^94^	Alverine Ccitrate 60 mg/simeticone 300 mg, 3 × 1/day, 4 weeks	207 patients Alverine citrate 60 mg/simeticone 300 mg, 205 patients placebo	IBS, Rome III	RCT	Abdominal pain VAS, abdominal discomfort, VAS	Significant decrease in VAS score in treatment groups, significant global symptom improvement in treatment groups	NA	Headache and nausea 2.9%, vertigo 1.9%
Remes Troche et al 2011 (Abstract)^95^	Pinaverium bromide 100 mg/simeticone 300 mg, 2 × 1/day, 12 weeks	127 patients Pinaverium bromide 100 mg/simeticone 300 mg, 128 patients placebo	IBS, Rome III	RCT	Abdominal pain, VAS	Significant improvement in VAS score in treatment group	NA	NA
Schmulson et al 2011^96^	Pinaverium bromide 100 mg/simeticone 300 mg, 2 × 1/day, 12 weeks	127 patients Pinaverium bromide 100 mg/simeticone 300 mg, 128 patients placebo	IBS, Rome III	RCT	Abdominal pain (VAS), abdominal volume calculated with a mathematical formula	Significant decrease in abdominal volume/pain in treatment group	NA	NA
Schmulson et al 2020^97^	Pinaverium bromide 100 mg/simeticone 300 mg, 2 × 1/day, 12 weeks	140 patients Pinaverium bromide 100 mg/simeticone 300 mg, 145 patients placebo	IBS, Rome III	RCT	Abdominal pain VAS, abdominal bloating VAS, Overall IBS symptom assesment	Significant decrease in abdominal pain/bloating VAS scores	NA	Acute pancreatitis in 1 patient in treatment group
Urgesi et al 2014^124^	Colinox 3 x 1/day (Simeticon+bacillus coagulans),	26 patients Colinox, 26 patients placebo	IBS, Rome III	RCT	Abdominal pain VAS, abdominal bloating VAS, abdominal discomfort VAS	Significant decrease in all VAS scores	NA

**Mebeverine hydrochloride**								
Connel AM et al 1965^102^	Mebeverine hydrochloride 100 mg 4 × 1/day, 12 weeks	20 patients Mebeverine hydrochloride, 20 patients placebo	IBS, Clinically	RCT	Global improvement of symptoms	Favours Mebeverine hydrochloride	Small sample size	Headache, dizziness, depression
Tasman-Jones C. et al 1973^103^	Mebeverine hydrochloride 100 mg 4 × 1/day, 4 weeks	12 patients Mebeverine hydrochloride, 12 patients placebo	IBS, Clinically	RCT	Abdominal pain and global improvement of symptoms	Favours Mebeverine hydrochloride	Small sample size	NA
Berthelot J et al 1981^104^	Mebeverine hydrochloride 100 mg 4 × 1/day, 8 weeks.	36 patients Mebeverine hydrochloride, 33 patients placebo	IBS, Clinically	RCT.	Global improvement of symptoms	Favors mebeverine		NA
Secco GB, 1983^107^	Mebeverine hydrochloride 135 mg 3 × 1/day, 4 weeks	15 patients Mebeverine hydrochloride, 15 patients placebo	IBS, Clinically	RCT	Relief of abdominal pain	NS	Small sample size	NA
Kruis W, 1986^105^	Mebeverine hydrochloride 100 mg 4 × 1/day, wheat bran, 16 weeks	40 patients Mebeverine hydrochloride, 40 patients placebo, 40 patients wheat bran	IBS,Clinical	RCT, (placebo and wheat bran controlled)	Global improvement of symptoms and abdominal pain	NS	Drop-out is very high (50%)	No side effects were reported
Enck P. 2005^106^	Mebeverine (posology is not reported), 16 weeks	Mebeverine hydrochloride, 40 patients placebo	IBS,Clinical	RCT	Global improvement of symptoms	NS		NA

PO, peppermint oil; EC, enteric-coated; MQS, overall methodological quality score; OSR, overall symptoms response; API, abdominal pain improvement; APR, abdominal pain resolution; APFI, abdominal pain frequency improvement; ADI, abdominal distension improvement; SFI, stool frequency improvement; SCI, stool consistency improvement; TCCT, total colon transit time; TPN, transit problems normalization, ASI, additional symptoms improvement; VAS, visual analog scale; IBS, irritable bowel syndrome;NA, not available, NS, not significant.

**Table 6.** Characteristics of studies analyzed in the meta-analysis of Bor S. et al^62^

Study (Country)	Sample size (pinaverium, placebo)	Mean age (years)	Proportion of female patients	Medication dosage (schedule)	Diagnostic criteria	Treatment duration (days)	MQS	Outcome assessed
Awad, 1995 (Mexico)	40 (20,20)	31	1	50mg (tid)	Rome I	21	8.11	OSR, API, ADI, SFI, SCI, ASI
Delmont, 1981 (France)	60 (30,30)	56	0.67	50mg (tid)	Clinical	28	5.72	OSR, API, ADI, TPN
Dubarry and Quinton, 1977 (France)	20 (10,10)	40	0.5	50mg (tid)	Clinical	6	4.86	APR
Levy, 1977 (France)	44 (22,22)	50	0.59	50mg (tid)	Clinical	15	6.63	OSR, API, ADI, TPN
Virat, 1987 (France)	78 (39,39)	44	0.51	50mg (tid)	Clinical	7	6.98	OSR, API, ADI, SFI
Zhang, 2011 (China)	28 (18,10)	40	0.5	50mg (tid)	Rome III	28	6.06	OSR, API, ADI, SFI, ASI
Zhao, 2004 (China)	60 (30,30)	37	0.5	50mg (tid)	Rome II	28	2.50	OSR, APR, ADI, TPN
Zheng, 2015 (China)	427 (218, 209)	37	0.47	50 mg (tid)	Rome III	28	9.24	OSR, APR, ADI, SFI, SCI

ADI, abdominal distension improvement; API, abdominal pain improvement; APR, abdominal pain resolution; ASI, additional symptoms improvement; MQS, overall Methodological Quality Score, OSR, overall symptoms response; SCI, stool consistency improvement; SFI, stool frequency improvement; TPN, transit problems normalization.

**Table 7.** Antispasmodics for Treatment of Functional Gastrointestinal Disorders in Children

Author, year	Intervention, duration	Participants	Diagnosis/ criteria	Study design	Primary outcome measures	Results	Limitations	Adverse Events (AE)
**Simeticon; Placebo controlled studies**								
Sethi KS 1988^93^	Simeticone (20-40 mg, 4 × 1/day) for 1 week, then crossed to placebo for the next 1 week	26 infants aged 1 week to 3 months with IC	No definition	RCT, cross-over	Number of daily crying episodes Intensity of crying attack	Simeticon was better in reducing the number of crying episodes and intensity of crying episode (*P *< .05)	No definition for IC No washout period	No AE
Danielsson B 1985^131^	Simeticone (28 mg before each meal) or placebo for 1 week, after a 3-day washout period crossed to other substance for the next 1 week	32 infants with IC, aged 2 to 8 weeks 5 infants withdrew after 2 to 7 dyas (2 receiving simeticone, and 3 placebo)	Crying in an otherwise healthy infant was diagnosed as IC	RCT, cross-over	Responders (defined as better or much better) Crying duration (hours/day) Time sleeping (hours/day) Number of feedings Number of stools	No difference in response rates No difference in daily crying duration, sleeping duration, number of feedings and stools	Randomization and blinding are not clear unclear Outcome measures are not clear No seperate results for the study periods 15% drop-out No colic definition	Not reported
Metcalf TJ 1994^92^	Simeticone (0.3 mL before each meal) or placebo for 1 week (range 3 days-10 days), followed by alternate substance for the second period	92 infants with IC, 2 to 8 weeks 9 infants excluded not related to study	Wessel criteria	RCT, cross-over	Responders (as improvement in symptoms, a 5-point scale was used) Crying duration	Improvement 28% with simeticone vs 37% with placebo (*P *> .05)	No washout period No seperate results for the study periods Blinding is not clear No fİxed duration for treatment	Not reported
**Simeticone; Studies used simeticone as control group**								
Savino F, 2007^137^	*L reuteri *(10^8^ live bacteria per day) vs simeticone (60 mg/d) for 4 weeks	90 infants, aged 21 to 90 days, with IC. 41 infants in probiotic group and 42 infants in simeticone group completed the study. No withdrawal related to the trial.	Wessel criteria	Randomised, open-label	Reduction of the daily mean crying time Response (defined as as a decrease in the daily average crying time out of 50% during the study)	On days 7, 14, 21, and 28 a significant reduction in daily crying time in probiotic group (*P *< .001) On day 28, 95% response rate in probiotic group vs 7% in simeticone group	Lack of true placebo group Open-label design	No AE
Alves JG, 2012^138^	Mentha piperita (liquid drops; 1 drop/kg) vs simeticone (2.5 mg/kg) daily for 7 days. After the first 7 days of study and a period of wash out for 3 days, all the children had their medication alternated and were followed for more 7 days	30 infants aged 8 to 56 days with IC. 3 of them excluded based on the exclusion criteria	Wessel criteria	RCT, cross-over	Number of daily episodes of colic and time spent crying	No difference between groups. 60% greatly or completely improvement in both groups	Lack of true placebo arm	No AE
Martinelli M, 2017^139^	A mixture of *Matricariae chamomilla L*., *Melissa officinalis L*. and tyndallized *Lactobacillus acidophilus* (Group A) vs *Lactobacillus reuteri* (Group B) and simeticone (60 mg, 2 × 1/day, Group C) for 4 weeks	180 infants, aged ≥2 weeks to 4 months, with IC. One patient of Group B and three patients of Group C were lost at follow-up	Rome II criteria	Prospective, multicentre, open-label, randomised controlled	Mean duration of crying time on day 28.	Significant decrease in mean crying time in Group A (*P *< .001) and Group B (*P *< .001) compared to Group C. No difference between groups A and B Response rate (defined as a decrease in the daily average crying time of 50% from baseline) 68.4% in Group C, 95% in Group A and 86.4% in Group B (*P *< .001)	Lack of true placebo Unblinded Open-label design	No AE
Raak C, 2019^142^	A homeopathic complex medicine (Enterokind) (during the acute phase 3 drops every hour, up to maximum of 6 × 1/day the dosage decreased to 3 drops, 3 × 1/day upon improvement), maximum 6 × 1/day vs simeticone (1 teaspoon 3-5 × 1/day) for 10 days	125 infants (74 in intervention group, 51 in simeticone group) aged ≤6 months with IC 13 patients in the Enterokind group and 7 patients in the control group violated the protocol (concomitant drug use)	No definiton	Prospective, multicenter, randomised, open-label, controlled.	Complaint Score (CS, consists of 9 complaints, maximum score 17) and Objective Symptoms Score (OSS, consist of 15 objective symptoms, maximum score 22).	Decrease in CS and OSS mean values in both groups, better in Enterokin group (*P *< .0001)	Unequal distribution of cases between groups Open-label design The use of non-validated scales High number of violation (although ITT analysis was done)	1 AE in Enterokind group (vomiting, unlikely related to substance) 4 AEs in simeticone group (2 allergic rashes- one of them was probably related to simeticone, 1 hard stool, and 1 viral infection
Piatek J, 2021^140^	Simeticon 100 mg/mL, 3-6 times 6 drops vs Multilac baby (9 probiotics and fructooligosaccharide) for 4 weeks	87 children (3-6 weeks) with colic (33 simeticon group)	Wessel criteria	Open-label, unequally ransomised (1:1.5)	Days of crying during the last 3 weeks, duration of evening crying, average number of crying phases per day	Significant reduction in the number of days of crying and the average duration of evening crying during the last 3 weeks. Multi-strain synbiotic is better than simethicon (*p*≤0.0001)	Medication is not blinded, use of parental diaries	No AE
Moldanado-Lobon JA, 2021^141^	Simeticone (4 x 20 mg), *B. breve *CECT7263 (2 × 10^8^ cfu/day), *B.breve* plus *L.fermentum *CECT5716 (both 1 × 10^8^ cfu/day), 4 weeks	150 infants, mean age 5.6±2.7 weeks	Rome III criteria	Open-label, randomised, controlled trial	Average crying duration/week.	Daily crying time decreased in all groups (*P *< .05)	18% withdrew, Open-label trial, relied on parent reports	Stomach pain (2 patients), constipation (4 patients), diaper rash (1 patient).
**Peppermint oil; Placebo controlled studies**								
Kline RM, 2001^145^	PMO (pH-dependent enteric coated capsule, <45 kg, 1 capsule (187 mg), 3 × 1/day >45 kg, 2 capsules 3 × 1/day, vs placebo (arachis oil) for 2 weeks	42 children (8-17 years) with IBS	Manning or Rome criteria	RCT	15-item Gastrointestinal Symptom Rating Scale (GSRS) Severity of symptoms scale Change in symptoms scale	Improvement in the severity of symptom scale 76% in PMO group and 19% in placebo group (*P *< .001) No significant change in GSRS No significant difference in improvement in the change of symptom scale (71.4% vs 42.9%, *P* = .06) OR for improvement 3.33 (95%CI 0.93- to 2.01)	Randomisation and blinding unclear No information about the method of analysis of the daily diaries	No AE
Asgarshirazi 2015^146^	PMO 187 mg 3 × 1/day (2 × 1/day for children<45 kg) vs synbiotic (Bacillus coagulans + FOS) vs placebo (1 mg folic acid) for 4 weeks	120 children (4-13 years) with FAP, functional dyspepsia, and IBS 34 patients in PMO group, 29 in synbiotic group and 25 in placebo group completed the study	Rome III criteria	Randomised, single-blind, parallel	Abdominal pain severity (1-10 scale), duration and frequency	Decrease in pain duration (*P *< .001), severity (*P *< .01), frequency (*P *< .001) greater compared with placebo Pain duration (*P* = 0.04) and severity (*P* = .013) better compared to synbiotic	Heterogeneity of the participants High drop-out rate (32 (26.7%) of the patients did not complete the study) No ITT analysis Single-blind	No AE
**Mebeverine; Placebo controlled study**								
Pourmoghaddas Z, 2014^149^	Mebeverine (135 mg, 2 × 1/day) vs placebo for 4 weeks	115 children (59 in mebeverine group, 56 in placebo group) (6-18 years) with FAP 44 and 40 patients in mebeverine group, and 43 and 39 patients in placebo group completed the 4-week and 12-week follow-up	Rome III criteria	RCT	Response (defined as ≥2 points reduction in the 6-point pain scale or “no pain”) at 4th and 12th week	Response rate (PP/ITT) in the treatment and placebo groups at 4 week 54.5%/40.6% vs 39.5%/30.3% (*P *> .05), at week 12; 72.7/54.2% vs 53.4/41% (*P *> .05)	28 (24.3%) of the patients dropped-out at 4th week and 31.3% at 12th week	Significantly more common dry mouth in the treatment group.
**Drotaverine; Placebo controlled study**								
Narang M, 2015^150^	Drotaverine (20 mg for children aged between 4-6 years, 40 mg for older ones) or placebo 3 × 1/day for 4 weeks	132 children (66 in each group) aged 4-12 years with RAP	Apley criteria*	RCT Stratification for ages 4-6 years and >6 years	Number of episodes of pain, and Number of pain-free days during 4 weeks	Reduction in number of episodes of abdominal pain (*P* = .01) No difference in pain-free days (*P* = 0.23) Parental satisfaction better in drotaverine group	Definition of RAP Short follow-up duration	Urticerial rash in 1 patient in drotaverine group (therapy discontinued) Macular rash in 4 patients in drotaverine group and in 1 patient in placebo group Vomiting, nausea, giddiness, diarrhea, headache, decreased appetite (no difference between groups)
**Hyoscine and its derivatives; Placebo controlled studies**								
Illingworth RS, 1955^153^	Methylscopalamine (0.05 mg, 4 × 1/day, before feeds) vs placebo (ascorbic acid) for an average of 17.9 days	40 babies, (mean age 4.5 weeks) with colic	Rhytmical screaming attacks in the evenings	RCT	Response; a scale ranging from +4 to -4 (+4 completely recovered, -4 much worse)	Improvement in 70% of the patients in the treatment group vs 80% in the placebo group. 25% of the babies worse or much worse in the treatment group	Unclear diagnostic criteria Indefinite treatment duration	No data
O’Donovan JC, 1979^154^	Group 1; 10 mg phenobarbital plus 0.25 mg homatropine methylbromide in 20% alcohol per 2.5 mL, Group 2; 10 mg phenobarbital in 20% alcohol per 2.5 mL, Group 3; 20% alcohol, 2.5 mL Group 4; placebo (water and artificial coloring) for 2 weeks.	110 babies (2 to 6 weeks) with infantile colic 27, 29, 28, and 26 patients in Groups 1, 2, 3, and 4, respectively	At least 90 minutes crying each day for a minimum of six of the preceding seven days	RCT	Response; a subjective scale ranging from -2 (crying increased by more than 90 min/day) to +2 (complete cessation of crying).	More than 70% of all patients improved No difference between groups	Subjective evaluation of the response	No data
**Cimetropium; Placebo controlled studies**								
Savino F, 2002^126^	Cimetropium (1.2 mg/kg at the onset of each crisis) vs placebo for 3 days	97 infants aged 15-60 days with IC 23 infants in each group completed the trial	Wessel criteria	RCT	Crying duration Response defined as crying time <15 minutes after administration of the compounds	Reduced crying duration with drug compared to placebo (*P *< .005) Response to drug 74% vs 33% with placebo (*P* = .0001) No difference in frequency of daily colic episodes	Randomization unclear No baseline characteristics No mention of total daily crying time	Sleepines more common in cimetropium group Others; meteorism, vomiting, inappetence, cutaneous reaction, constipation sleepiness, restlessness, respiratory distress, apnea
**Cimetropium; Dose comparison**								
Gomirato, 1989^157^	Cimetropium 1.2 mg/kg vs cimetropium 2.0 mg/kg for 2 weeks. Infants received the first treatment for 7 days, then crossed to the other treatment group for the next seven days	40 infants of average age 4.4 weeks (range not reported) with infantile colic	Wessel criteria	Parallel-group study	Reduction in the number of crying episodes and in their duration	No difference in crying duration (favored in higher dose group) and the frequency of crying episodes (favored in low dose group) No difference between groups regarding improvement in symptoms	The two groups differed at baseline No information on the method of random sequence, blinding of parents and outcome measures	Four infants (20%) receiving the higher dosage had constipation.

## Data Availability

The data that support the findings of this study are available on request from the corresponding author.
